# Molecular and Functional Alterations in the Cerebral Microvasculature in an Optimized Mouse Model of Sepsis-Associated Cognitive Dysfunction

**DOI:** 10.1523/ENEURO.0426-23.2024

**Published:** 2024-09-26

**Authors:** Paulo Ávila-Gómez, Yuto Shingai, Sabyasachi Dash, Catherine Liu, Keri Callegari, Heidi Meyer, Anne Khodarkovskaya, Daiki Aburakawa, Hiroki Uchida, Giuseppe Faraco, Lidia Garcia-Bonilla, Josef Anrather, Francis S. Lee, Costantino Iadecola, Teresa Sanchez

**Affiliations:** ^1^Department of Pathology and Laboratory Medicine, Weill Cornell Medicine, New York, New York 10065; ^2^Department of Psychiatry, Weill Cornell Medicine, New York, New York 10065; ^3^Department of Neuroscience, Feil Family Brain and Mind Research Institute, Weill Cornell Medicine, New York, New York 10065

**Keywords:** blood–brain barrier, cerebral microvascular dysfunction, cognitive decline, dementia, inflammation, systemic inflammatory response syndrome

## Abstract

Systemic inflammation has been implicated in the development and progression of neurodegenerative conditions such as cognitive impairment and dementia. Recent clinical studies indicate an association between sepsis, endothelial dysfunction, and cognitive decline. However, the investigations of the role and therapeutic potential of the cerebral microvasculature in sepsis-induced cognitive dysfunction have been limited by the lack of standardized experimental models for evaluating the alterations in the cerebral microvasculature and cognition induced by the systemic inflammatory response. Herein, we validated a mouse model of endotoxemia that recapitulates key pathophysiology related to sepsis-induced cognitive dysfunction, including the induction of an acute systemic hyperinflammatory response, blood–brain barrier (BBB) leakage, neurovascular inflammation, and memory impairment after recovery from the systemic inflammation. In the acute phase, we identified novel molecular (e.g., upregulation of plasmalemma vesicle-associated protein, PLVAP, a driver of endothelial permeability, and the procoagulant plasminogen activator inhibitor-1, PAI-1) and functional perturbations (i.e., albumin and small-molecule BBB leakage) in the cerebral microvasculature along with neuroinflammation. Remarkably, small-molecule BBB permeability, elevated levels of PAI-1, intra-/perivascular fibrin/fibrinogen deposition, and microglial activation persisted 1 month after recovery from sepsis. We also highlight molecular neuronal alterations of potential clinical relevance following systemic inflammation including changes in neurofilament phosphorylation and decreases in postsynaptic density protein 95 and brain-derived neurotrophic factor, suggesting diffuse axonal injury, synapse degeneration, and impaired neurotrophism. Our study serves as a standardized mouse model to support future mechanistic studies of sepsis-associated cognitive dysfunction and to identify novel endothelial therapeutic targets for this devastating condition.

## Significance Statement

The limited knowledge of how systemic inflammation contributes to cognitive decline is a major obstacle to the development of novel therapies for dementia and other neurodegenerative diseases. Clinical evidence supports a role for the cerebral microvasculature in sepsis-induced neurocognitive dysfunction, but the investigation of the underlying mechanisms has been limited by the lack of standardized experimental models. Herein, we optimized a mouse model that recapitulates important pathophysiological aspects of sepsis-induced cognitive decline and identified key alterations in the cerebral microvasculature associated with cognitive dysfunction. Our study provides a reliable experimental model for mechanistic studies and therapeutic discovery of the impact of systemic inflammation on cerebral microvascular function and the development and progression of cognitive impairment.

## Introduction

Systemic inflammation has been associated with the development and progression of several neurodegenerative conditions, including cognitive impairment and dementia. Up to 50% of patients who recover from severe systemic inflammation (i.e., systemic inflammatory response syndrome, SIRS, and sepsis) develop persistent cognitive impairment and accelerated dementia (Holmes et al., 2009; Iwashyna et al., 2010; Widmann and Heneka, 2014; Annane and Sharshar, 2015; Chou et al., 2017; Mostel et al., 2019; Beltran-Garcia et al., 2020; Bettcher et al., 2021; Hampshire et al., 2021; Sipilä et al., 2021; Y. H. Liu et al., 2021; Manabe and Heneka, 2022). The cellular and molecular mechanisms leading to neurocognitive dysfunction in sepsis survivors are not well understood, and further research is needed to develop novel therapeutic strategies to prevent and treat this devastating condition.

Increasing clinical evidence indicates that the endothelium and the blood–brain barrier (BBB) are profoundly affected during sepsis and SIRS and could play an important role in the development of cognitive impairment (Widmann and Heneka, 2014; Gust et al., 2017; Thakur et al., 2021; Greene et al., 2024). The cerebrovascular endothelium, a key housekeeper of the BBB and neurovascular homeostasis, constitutes a promising therapeutic opportunity. Thus, further examination of the molecular and cellular mechanisms leading to and resulting from BBB and cerebral microvascular dysfunction is required to broaden our understanding of how systemic inflammatory factors signal via the cerebral microvasculature to alter cognitive function and identify novel therapeutic targets. However, these investigations have been hampered by the lack of standardized experimental models to determine the alterations in the cerebral microvasculature and cognition induced by systemic inflammation. To this end, in the present study, we have established a mouse model of endotoxemia that recapitulates key aspects of the pathophysiology of sepsis-induced cognitive dysfunction including the induction of an acute systemic hyperinflammatory response, loss of BBB integrity, and neurovascular inflammation, followed by alterations in cognitive function in the subacute and chronic phases, after recovery from systemic inflammation. We have validated a noninvasive and effective scoring system to assess the clinical signs of the systemic inflammatory response. In addition, we have identified key molecular (i.e., induction of the caveolar protein plasmalemma vesicle-associated protein, PLVAP, and the antifibrinolytic plasminogen activator inhibitor, PAI-1) and functional alterations in the cerebral microvasculature (i.e., albumin and small-molecule BBB leakage and intra-/perivascular fibrin/fibrinogen deposition), along with perturbations in neuronal molecular markers associated with cognitive dysfunction of potential clinical relevance, including changes in neurofilament, postsynaptic density protein 95 (PSD-95), and brain-derived neurotrophic factor (BDNF). This work will permit future investigations of the mechanisms underlying sepsis-induced cognitive dysfunction, particularly, the role of the cerebral microvasculature, and provide a reliable model for the therapeutic discovery of novel vasoprotective agents in cognitive impairment and other neurodegenerative pathologies.

## Materials and Methods

### Animals and LPS treatments

All animal experiments were approved by the Institutional Animal Care and Use Committee. Approximately 152 C57/BL6 mice (8–10 weeks, male, Jackson Laboratory) were used for this study. Animals were housed in stable environmental conditions (23°C, 40% relative humidity, and 12 h light/dark cycle), with access to food and water *ad libitum*.

**Figure 1.**
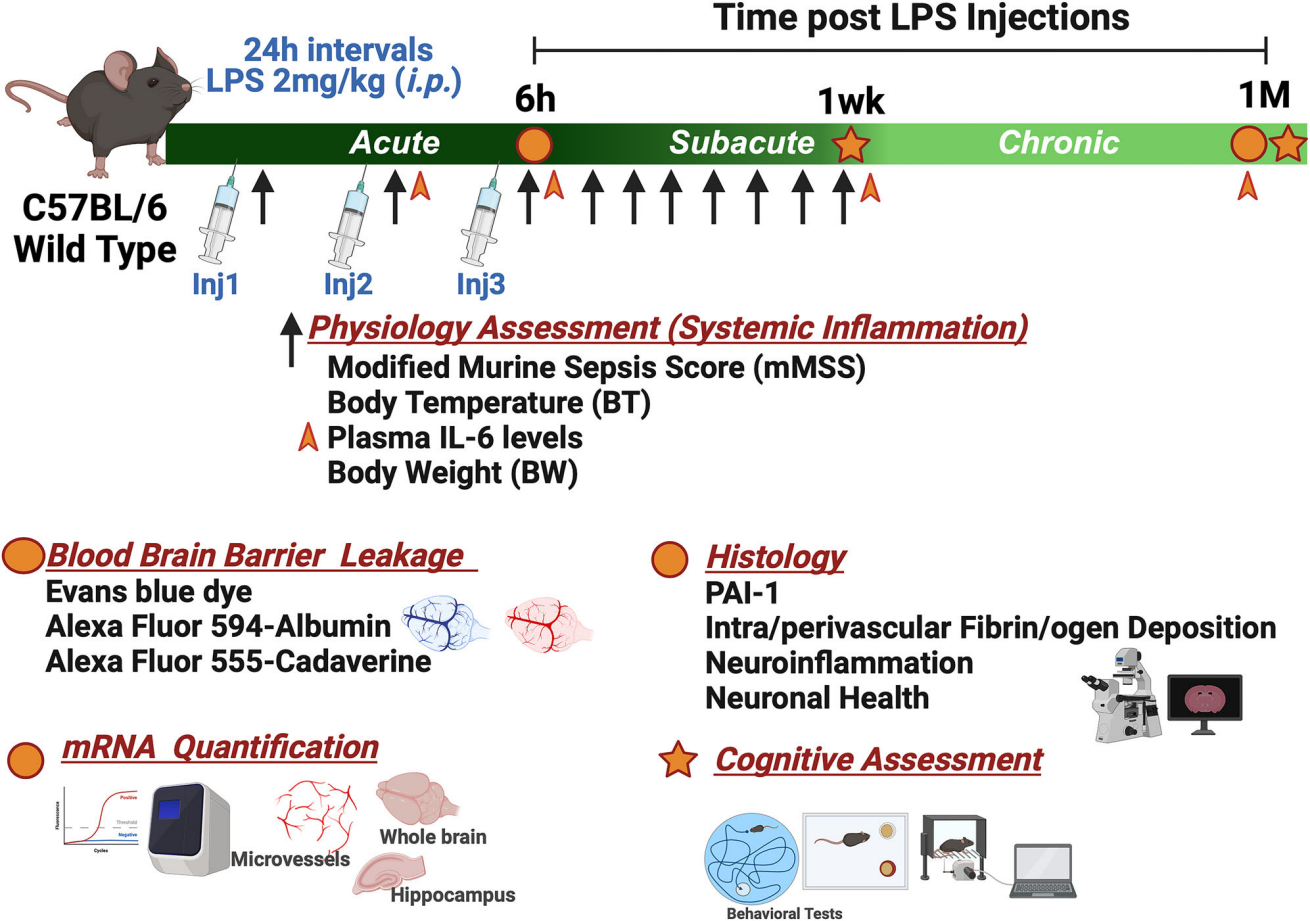
Experimental timeline and subsequent assessments following LPS injections. LPS was administered for 3 consecutive days. Physiological assessment of the clinical signs of the systemic inflammatory response was conducted 6 h after each injection and continued daily for 1 week (black arrows). Blood collection for determination of plasma IL-6 levels was conducted 6 h after the second and third LPS or saline injections and 1 week and 1 month afterward (orange arrowheads). Behavioral testing was performed after recovery from the systemic inflammatory response, i.e., 1 week and 1-month post-LPS injections. Blood–brain barrier (BBB) permeability determination to albumin and small molecules [Evans blue dye (EBD) extravasation assay, histological determination of Alexa Fluor 594 albumin and Alexa Fluor 555 cadaverine leakage], RNA quantification (qRT-PCR analysis), and immunofluorescence imaging were conducted 6 h (acute phase) and 1 month after last LPS injection (chronic phase).

LPS endotoxin [*Escherichia coli* O111:B4 (Sigma-Aldrich L4391)] was prepared as a stock solution at 2 mg/ml in normal saline (0.9% w/v) and further diluted in saline for a 200–250 μl injection per mouse (at a dose of 2 mg/kg) on the day of use. We optimized LPS treatments at 2 mg/kg, i.p., per mouse body weight using a three-injection routine at 24 h intervals to induce severe and sustained systemic inflammation. In this model, the mortality rates were ∼10% in the first 3 d following LPS injection (acute phase) and <5% in the subacute (Days 4–7) and chronic (Day 7–1 month) phases. Tissues were harvested immediately following euthanasia and flash-frozen before storage and molecular analyses (Fig. 1).

### Physiological assessment of the clinical signs of the systemic inflammatory response

Mice were assessed every 6 h post-LPS injection and daily during the first week after LPS administrations to determine murine sepsis scoring and changes in temperature and body weight. Plasma levels of IL-6, a reliable biomarker of systemic inflammation, were also determined. Five to six mice per group (saline and LPS) were used for these studies.

#### Murine sepsis scoring with nesting behavior (MSS)

Systemic inflammation was induced by intraperitoneal injection of 2 mg/kg LPS at 24 h intervals over 3 d (Fig. 1). To assess the clinical signs of systemic inflammation, we designed a multiparametric assessment scale that combines the murine sepsis score (MSS; Shrum et al., 2014) and nesting behavior (Deacon, 2006) rating scale [modified MSS (mMSS)]. The assessed parameters included features such as appearance (piloerection, puffiness), level of consciousness (posture impairment, social engagement), activity (exploratory behavior, movement speed), response to stimulus (reaction time to sound and touch stimuli), eye condition (presence of secretions or closed eyelids), respiratory rate (presence of apneas, slowed breathing pace), and nesting behavior, which are affected following inflammation and are reminiscent of the sepsis phenotype. For the nesting assessment, nesting pads (Nestlets, Ancare) were placed in the mice cages after every saline or LPS injection. Healthy mice quickly tear the pads to smithereens and build a nest from scratch, whereas septic mice’s ability to build the nest decreases, leading to larger portions or even intact nesting pads. A new nesting pad was placed every day. This scoring system is described in detail in Table 1.

**Table 1.** Assessment parameters of modified murine sepsis score (mMSS)

Score	0	1	2	3	4
Appearance	Smooth	Patches of hair piloerected	Majority of the back is piloerected	Mouse appears “puffy”	Mouse appears emaciated
Level of consciousness	Active	Still active but avoids standing upright	Slowed, still ambulant	Impaired, only provoked move with a tremor	Severely impaired, stationary with possible tremor
Activity	Normal	Slightly suppressed	Suppressed	No activity	No activity and tremors, particularly in the hind legs
		Moving around the bottom of the cage	Stationary with occasional exploration		
Response to stimulus	Immediately to an auditory stimulus or touch	Auditory: slow or no response	Auditory: no response	Auditory: no response	Auditory: no response
		Touch: strong (moves to scape)	Moderate (moves a few steps)	Touch: mild (no locomotion)	Touch: little or no response
Eyes	Open	Eyes not fully open, possibly with secretions	Eyes at least half closed, possibly with secretions	Eyes half closed or more, possibly with secretions	Eyes closed or milky
Respiration rate	Normal, rapid, not quantifiable by the eye	Slightly decreased (not quantifiable by the eye)	Moderately reduced (quantifiable by the eye)	Severely reduced (0.5 s between breaths)	Extremely reduced (>1 s between breaths)
Nesting behavior	Normal, fully scratched, and nested	The majority remains fluffy with some parts intact	Small piece of the nest, partially fluffy	Large portion remains intact	Largely untouched

#### Changes in temperature and body weight

Body weight measurements were recorded using a portable weighing balance. Body temperature was recorded using a rectal thermal probe.

#### Plasma levels of IL-6

Blood was obtained via submandibular vein puncture using lancets and collected into 0.5 M EDTA-coated capillaries, 6 h after the second and third LPS or saline injections and 1 week and 1 month after the last LPS or saline injection. Plasma was then obtained by centrifugation of the collected samples for 15 min at 1,500 × *g* and stored at −20°C until further analyses. Plasma IL-6 levels were then determined using a commercially available mouse IL-6 Quantikine ELISA Kit (R&D Systems, M6000B). Five to six mice per group (saline and LPS) were used for plasma collection.

### Determination of BBB leakage

#### Evans blue dye (EBD) extravasation assay

BBB leakage to albumin was assessed by EBD extravasation assay in saline- and LPS-injected mice at 6 h and 1 month after the third injection, as previously described (Kim et al., 2015). Five to eight animals per group were used for these studies. Two percent of EBD (wt/vol in PBS) at 0.2 mg/g was injected into the right jugular vein and allowed to circulate for 2 h. Under deep anesthesia, mice underwent transcardial perfusion with PBS (+5 mM EDTA) via a 23-gauge butterfly needle through the left ventricle until a clear fluid was obtained from the right atrium (∼20 ml). The brains were removed, and the olfactory bulb and cerebellum were cut off. The resulting brain hemispheres were cut in half with half taken for EBD measurement and the other half for RNA extraction. The hemispheres were weighed, and EBD was extracted with 50% trichloroacetic acid (TCA) as previously described (Zhang et al., 2013; Kim et al., 2015; Yanagida et al., 2017). Briefly, one brain hemisphere was weighed and then homogenized in 1 ml of 50% TCA (weight/volume) and subjected to centrifugation at 15,000 × *g* for 15 min. This centrifugation was repeated an additional time to remove the leftover debris. The concentration of EBD in the supernatant was measured in triplicates using fluorescence spectroscopy (620_ex_/680_em_).

#### Histological determination of albumin and cadaverine BBB leakage

Albumin and small-molecule (cadaverine) BBB leakage were determined in separate groups of mice, treated with saline or LPS, at 6 h or 1 month after the third injection (3–4 animals per group). Alexa Fluor 594 albumin (5 mg/ml, Thermo Fisher Scientific) solution in saline was injected into the right jugular vein. After allowing to circulate for 2 h, mice were transcardially perfused as previously described with cold PBS/EDTA followed by 4% PFA and further processed for histology. Likewise, 1.5 mg/ml of Alexa Fluor 555 cadaverine was injected at a 6 µg/g dose using the same approach.

### Cerebral microvessel isolation

Cerebral microvessels were isolated from saline- and LPS-injected mice at 6 h and 1 month after the third injection, as previously described (Y-K. Lee et al., 2019). Five animals per group were used for microvessel isolation. To minimize cell activation, all procedures were conducted in a cold room. Ipsilateral cortices were homogenized with MCDB131 medium (Thermo Fisher Scientific, 10372019) with 0.5% fatty acid-free BSA (MilliporeSigma, 126609). The homogenate was centrifuged at 2,000 × *g* for 5 min at 4°C. The pellet was suspended in 15% dextran (molecular weight, ∼70 kDa, MilliporeSigma, 31390) in PBS and centrifuged at 10,000 × *g* for 15 min at 4°C. The pellet containing the microvessels was resuspended in MCDB131 with 0.5% fatty acid-free BSA and centrifuged at 2,000 × *g* for 10 min at 4°C.

### mRNA quantification, cDNA synthesis, and quantitative PCR (qPCR)

Six hours or 1 month after the third LPS or saline injections, RNA was extracted from the whole brain (3–5 animals per group), isolated cerebral microvessels (5 animals per group), or hippocampus (3–5 animals per group) using TRIzol (Thermo Fisher Scientific) and the Qiagen RNeasy Mini Kit (Qiagen) as previously described (Y-K. Lee et al., 2019). cDNA was produced using a Verso Reverse Transcriptase (RT) kit with Random Hexamer Primers (Thermo Fisher Scientific) and diluted with nuclease-free water. SYBR Green with ROX dye (Quantabio)–based qPCR was performed to determine the relative expression levels of target genes, Claudin-5 (Cldn5), Plvap, Caveolin-1 (Cav1), Serpine-1, and Bdnf. qPCR reactions were run in duplicates or triplicates (depending on the amount of RNA/cDNA available) on a 96-well plate loaded onto an ABI-7500 Sequence Detection System PCR machine (Applied Biosystems), and the averages of the duplicates/triplicates Ct values were calculated. Ct values of target genes were normalized to expression levels (Ct values) of hypoxanthine phosphoribosyltransferase (Hprt) RNA, as ΔCt values. For saline and LPS samples, the relative gene expression levels were expressed as ΔΔ Ct values by subtracting the average ΔCt values of the saline group from each ΔCt value. Fold change in target gene expression was calculated by comparing the 2^−ΔΔCt^ values of the LPS samples and saline samples. The primer sequences and gene ID numbers are presented in Table 2.

**Table 2.** Primer sequences and gene ID numbers

Target genes (*Mus musculus*)	qPCR primer (5′→3′ orientation)	Ref Seq ID
Cldn5	FP-CCCAGTTAAGGCACGGGTAG	NM_013805.4
	RP-GGCACCGTCGGATCATAGAA	
Plvap	*FP*-AGCCAGGTGGTTGGACTATC	NM_032398.2
	*RP*-TAGCGGCGATGAAGCGATTA	
Cav1	FP-GACCCCAAGCATCTCAACGA	NM_007616.4
	RP-AAATGCCCCAGATGAGTGCC	
Serpine-1 (PAI-1)	*FP*-GGCACAGTGGCGTCTTCCT	NM_008871.2
	*RP*-TGCCGAACCACAAAGAGAAAG	
Bdnf	FP-TAT AAA TGA AGT TTA TAC AGT ACA GTG	NM_007540.4
	RP-AAC ATT ATC GAG GAA TGT AAT GCA G	
Hprt	FP-ACCTCTCGAAGTGTTGGATACAG	NM_013556.2
	RP-TTCACTAATGACACAAACGTGATTC	

### Barnes circular maze

One week after the third LPS or saline injection, mice were subjected to the Barnes maze test. Nine to 10 animals per group were used. The Barnes circular maze consisted of a circular open surface (90 cm in diameter) elevated to 90 cm by four wooden legs (O’Leary and Brown, 2013; Faraco et al., 2019). There were 20 circular holes (5 cm in diameter) equally spaced around the perimeter and positioned 2.5 cm from the edge of the maze. No wall and no intramaze visual cues were placed around the edge. A wooden plastic escape box (11 × 6 × 5 cm) was positioned beneath one of the holes. Three neon lamps and a buzzer were used as aversive stimuli. The ANY-Maze tracking system (Stoelting) was used to record the movement of mice in the maze. Extra-maze visual cues consisted of objects within the room (table, computer, sink, door) and the experimenter. Mice were tested in groups of 7–10, and between trials, they were placed into cages, which were placed in a dark room adjacent to the test room for the intertrial interval (30–60 min). No habituation trial was performed. The acquisition phase consisted of three consecutive training days with three trials per day with the escape hole located at the same location across trials and days. On each trial, a mouse was placed into a start tube located in the center of the maze, the start tube was raised, and the buzzer (white noise) was turned on until the mouse entered the escape hole. After each trial, mice remained in the escape box for 60 s before being returned to their cage. Between trials, the maze floor was cleaned with 10% ethanol in water to minimize olfactory cues. For each trial, mice were given 3 min to locate the escape hole, after which they were guided to the escape hole or placed directly into the escape box if they failed to enter the escape hole. The parameters recorded for learning performance were below (1) the latency to locate (primary latency) and (2) the distance traveled before locating the escape hole (Faraco et al., 2019). On Day 4, the location of the escape hole was moved 180° from its previous location, and two trials per day were performed. ANY-Maze v5.3 was used for the collection and analysis of the behavioral data.

### Novel object recognition (NOR)

One month after the third LPS or saline injection, mice were subjected to the NOR test (12–14 per group). The NOR task was conducted under dim light in a plastic box with dimensions 29 cm × 47 cm × 30 cm high. Stimuli consisted of plastic objects that varied in color and shape but had similar sizes (Antunes and Biala, 2012; Cohen and Stackman, 2015; Grayson et al., 2015). A video camera mounted on the wall directly above the box was used to record the testing session for offline analysis. Mice were habituated to the testing environment and chamber for 24 h prior to testing. Thereafter, mice were placed in the same box in the presence of two identical sample objects and were allowed to explore for 5 min. After an intersession interval of 24 h, one of the two objects was replaced by a novel object, and mice were returned to the same box and allowed to explore for 5 min (Faraco et al., 2019). Recognition memory using this paradigm has been shown to depend on the integrity of both the hippocampus and cortex (Antunes and Biala, 2012; Cohen and Stackman, 2015). Exploratory behavior was then assessed manually by a researcher blinded to the treatment group. Exploration of an object was defined as the mouse sniffing the object or touching the object while looking at it. Placing the forepaws on the objects was considered an exploratory behavior but climbing on the objects was not. The discrimination ratio was calculated by dividing the difference in the amount of exploration time between novel and familiar objects by the total exploration time. ANY-Maze v5.3 was used for the collection and analysis of the behavioral data.

### Associative fear conditioning

A battery of behavioral protocols was carried out to assess fear response 1 month after the third LPS or saline injection (12–29 mice/group). Fear test was carried out using established protocols (Meyer et al., 2019). Protocols were carried out in standard conditioning chambers (Med Associates). The chambers (30 × 24 × 27 cm) consisted of clear acrylic front and back walls, aluminum sides, stainless steel grid floors, and a clear acrylic top. Each chamber was outfitted with a speaker located 13 cm above the grid floor, used to present the auditory conditioned stimulus. Delivery of a footshock through the grid floor served as the aversive unconditioned stimulus. For context setup, a chamber with LED Stimulus Light (50 lux) mounted 18 cm above the grid floor to provide background illumination containing olfactory cues (peppermint, 1/1,000 in ethanol) was used. Each chamber was enclosed in a sound-attenuating cubicle (71 × 59 × 56 cm) with a 28 V DC exhaust fan to provide airflow and background noise. VideoFreeze® software (Med Associates) was used to control experiments, and all trials were videotaped for analysis. Freezing, defined as the absence of visible movement except that required for respiration, was quantified using the VideoFreeze® software set at a motion threshold of 18 units for automatic scoring.

#### Fear conditioning

Mice were acclimated to the conditioning chamber for 2 min prior to five tone presentations (5 kHz, 80 dB, 20 s duration) that coterminated with a footshock (0.5 mA, 1 s duration). The intertrial interval (ITI) between the tone–shock pairings was set at 1 min. Mice remained in the conditioning chamber for 1 min after the final tone–shock pairing before being returned to their home cages. The baseline motion was recorded during acclimation of the first 2 min, and then the percentage time spent freezing during each tone (20 s) was calculated, providing an index of cued fear learning.

#### Contextual fear recall test

Mice were tested for contextual fear 24 h after fear conditioning. Mice were returned to the context setup for 5.5 min during which no tones or shocks were presented. Upon completion of the contextual fear test, mice were returned to their home cages. The percentage of time spent freezing across the first 2 min of the entire 5.5 min session was calculated, providing an index of contextual fear memory.

### Immunofluorescence

Six hours or 1 month after the third LPS or saline injections, mice were perfused with cold PBS under deep anesthesia and subsequently with 4% PFA in PBS solution. Three to five mice per group were used for these histological analyses. The brains were removed, postfixed with 4% PFA for 24 h, transferred to 30% sucrose solution in PBS, embedded in optimal cutting temperature (OCT) compound, frozen on liquid nitrogen, and placed in a −80°C freezer for storage. Ten-micrometer-thick sections were cut on a cryostat (Leica Microsystems) through +0.5 to −2.6 mm from the bregma. Sections were washed three times with PBS and then blocked with blocking solution (5% bovine serum albumin, 0.8% skim milk, and 0.3% Triton X-100 in TBS) for 1 h; incubated with the specified primary antibodies in blocking solution overnight at 4°C, followed by the appropriate secondary antibodies and 4′,6-diamidino-2-phenylindole (DAPI) for 1 h at room temperature; and were mounted onto slides. The cerebral microvasculature was identified by immunofluorescence for the endothelial-specific marker, glucose transporter 1 (Glut1), as previously described (Callegari et al., 2023). The following antibodies were used in this study: anti-GLUT1, Alexa Fluor 488–conjugated (rabbit polyclonal, from MilliporeSigma, 1:100), anti-SERPINE-1 (rabbit polyclonal, from Innovative Research, 1:100), anti-ionized calcium-binding adaptor molecule 1 (IBA1, rabbit polyclonal, from Wako, 1:100), anti-glial fibrillary acidic protein (GFAP, rabbit polyclonal, from Abcam, 1:100), anti-BDNF (rabbit monoclonal, from Abcam, 1:100, which recognizes pro-BDNF and biologically active BDNF isoforms), anti-hyperphosphorylated neurofilament heavy chain (pNF, mouse monoclonal, clone SMI 31P, from BioLegend, 1:100), purified anti-neurofilament H (NF-H), nonphosphorylated antibody, clone SMI32 (801701, BioLegend, 1:100), recombinant Alexa Fluor 647 anti-P2Y12 antibody (EPR26298-93; ab308432, Abcam), anti-fibrinogen (rabbit polyclonal ab34269, Thermo Fisher Scientific), and recombinant anti-PSD-95 (EPR23124-118; ab238135, Abcam).

### Image acquisition and analysis

Images were acquired on an Agilent BioTek Lionheart FX automated microscope (20× magnification objective; Agilent) and quantified using BioTek Gen5 software for pNF (SMI31), SMI32, P2Y12, IBA1, PSD-95, and fibrinogen and QuPath software for PAI1, GFAP, and BDNF. Images for Alexa Fluor 594 albumin and Alexa Fluor 555 cadaverine analysis were acquired using a Leica Stellaris confocal microscope (40× magnification objective) and analyzed using QuPath software. For all analyses, images were acquired from two sections per mouse brain, one anterior (+0.5 to −0.3 mm from the bregma) and one posterior (−1.8 to −2.6 mm from the bregma). A total of 6–10 brain sections from three to five mice were quantified. For analysis using BioTek Gen5, 10–12 regions of interest (ROI) from the cortex and 3–6 ROI from the hippocampus per mouse brain were acquired and quantified. For analysis using QuPath, whole-brain images were acquired, and 10–12 (ROI) from the cortex and 18–20 small ROI (4–6 ROI for BDNF) from the hippocampus were quantified per mouse brain (Extended Data Fig. 3-2). The vessel area on each ROI was automatically detected using GLUT1 (endothelial-specific marker) staining and QuPath's built-in cell segmentation algorithms as previously described (Callegari et al., 2023). Albumin puncta were quantified within the vessel and in a 10 µm region around the vessel and normalized by vessel area. PAI-1 total intensity in vessel detected area, fibrinogen positive area in vessel detected area, or IBA1, P2Y12, GFAP, BDNF, PSD-95, SMI31, SMI32, and cadaverine positive area detected by thresholding on each ROI were quantified and normalized by the mean value of the ROI of saline-injected mice. In P2Y12+ regions, the IBA1+ integrated density was used as a thresholder to detect P2Y12+/IBA1+ regions. All quantified data were exported to Excel and Prism 9 for further analysis.

### Statistical analysis

For EBD leakage assay, qRT-PCR, image analysis quantification, and behavior assessment statistical analyses were conducted using GraphPad Prism 9 software. All values reported are mean ± SEM.

For comparisons between two groups, an unpaired *t* test was used. For data involving time series, a two-way analysis of variance (ANOVA) followed by Sidak's multiple-comparisons test was used. For comparisons among the three groups (control group, 6 h after the final LPS administration, and 1 month after), a one-way ANOVA followed by Tukey's multiple-comparisons test was used. *p* < 0.05 was defined as statistically significant (*p < 0.05, **p < 0.005, ***p < 0.0005, ****p < 0.0001). A statistical table for all analyses is provided in Extended Data Table 1.

10.1523/ENEURO.0426-23.2024.t1-1 Table 1 Table supporting Figures 2, 3, 4, 5 and 6. Statistical table for all analyses. Download Table 1, XLSX file.

## Results

### Characterization of the endotoxemia-induced mouse model of systemic inflammation

**Figure 2.**
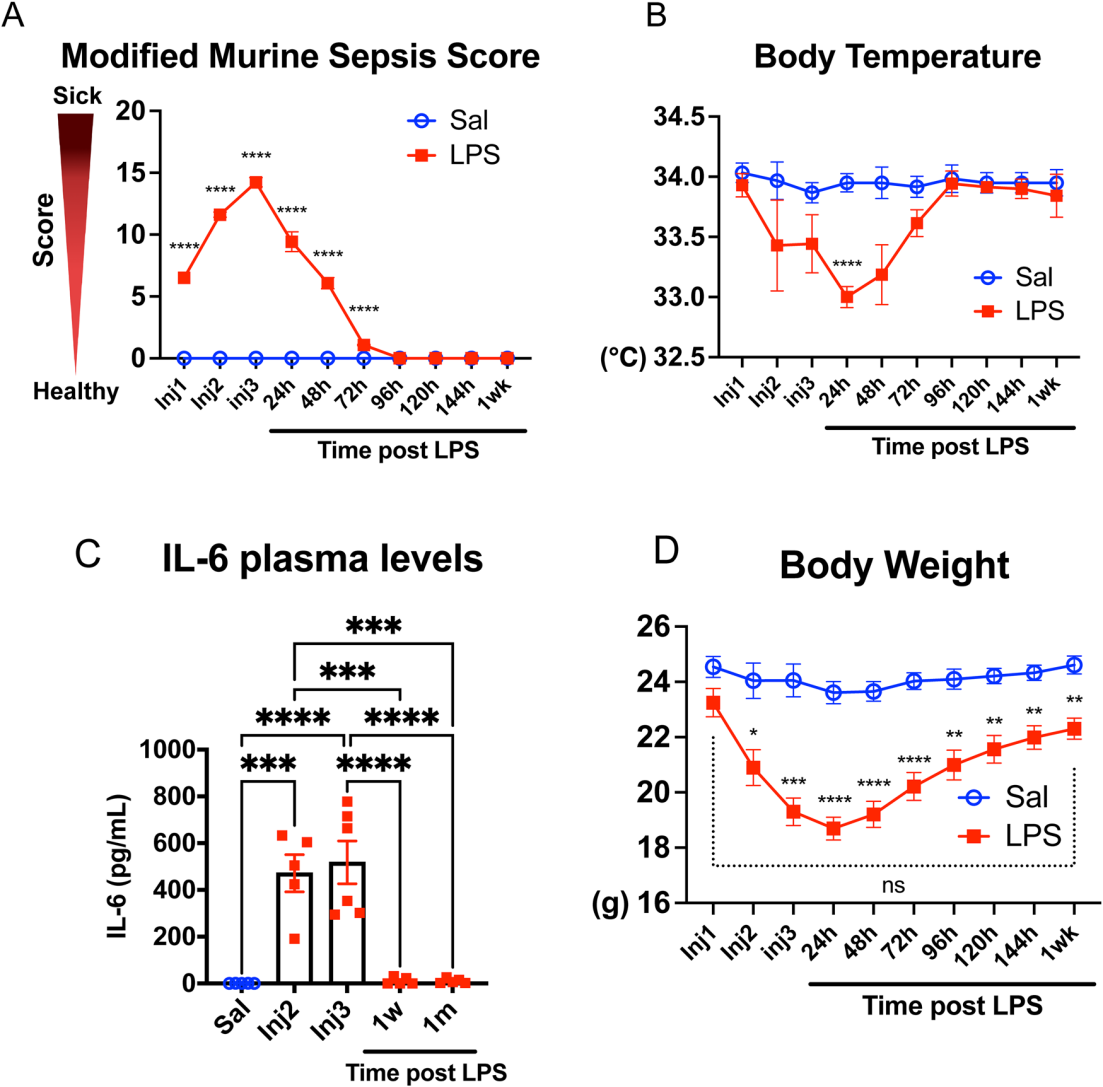
LPS injections induce an acute systemic inflammatory response that resolves within 72–96 h. Following LPS injections, assessments of the modified murine sepsis score (mMSS), body temperature, IL-6 plasma levels, and body weight were conducted to evaluate physiological changes and gauge the severity of systemic inflammation. ***A***, The mMSS score surged immediately following the initial injection, reaching its peak after the third dose. Following LPS treatment, the mMSS score rapidly diminished, matching the saline control levels from 96 h onwards. ***B***, Body temperature exhibited a decline during LPS treatments and returned to baseline levels 96 h after the last LPS dose. ***C***, IL-6 plasma levels (pg/ml) were significantly increased following LPS injection and returned to baseline levels at 1 week after LPS administration (*N* = 5–6/group). ***D***, Body weight demonstrated a pronounced decrease during the LPS injection period, began recovery 24 h after the final LPS administration, and nearly reached baseline 1 week after the last injection. The mean of individual value ± SEM is shown. ***A***, ***B***, ***D***, *N* = 7 saline, *N* = 13 LPS. *p < 0.05, **p < 0.005, ***p < 0.0005, ****p < 0.0001, two-way ANOVA followed by Sidak's multiple-comparisons test. Please refer to Extended Data Figure 2-1 for extended data supporting this figure (changes in the individual parameters contributing to the overall mMSS).

We established a mouse model of endotoxemia to recapitulate important clinical and neurological features of systemic inflammatory response syndrome and sepsis, including the induction of a hyperinflammatory response via the activation of pathogen recognition receptors, as well as key aspects of the pathophysiology of cognitive dysfunction induced by severe systemic inflammation including BBB leakage, neuroinflammation, and memory impairment (Fig. 1). We administered LPS treatments at 2 mg/kg per mouse body weight using a three intraperitoneal injection protocol at 24 h intervals to induce a sustained systemic inflammatory response (Fig. 2). We next determined the clinical signs of systemic inflammation using a multiparametric assessment scale, i.e., murine sepsis scores, MSS, and changes in body temperature. Our modified MSS (mMSS) included a previously described scoring system (Shrum et al., 2014) and a nesting behavior score (Deacon, 2006). Mice were scored on six variables including grooming, activity, consciousness, eye secretions, respiratory rate, stimulus response, and nesting behavior (Table 1). LPS administration caused a rapid and acute response in dosed mice, as reflected by an immediate increase in mMSS following the initial injection. The mMSS continued to increase with every injection peaking after the third dose and returned to normal baseline 72–96 h postfinal injection (Fig. 2*A*). Changes in the individual parameters are shown in Extended Data Figure 2-1. Regarding body temperature, LPS-dosed mice showed a 0.5°C decrease in body temperature after the second injection. At 24 h following the third LPS administration, dosed mice showed a significant reduction (33.0°C) in body temperature compared with the saline group (34.0°C). In a similar pattern to mMSS, animals’ body temperature slowly recovered beyond 24 h and reached baseline levels at 72–96 h (Fig. 2*B*). These changes in mMSS and body temperature suggest that the acute systemic inflammatory response induced by LPS resolved near 72 h after last injection. We also confirmed the development and resolution of the systemic inflammatory response by measuring plasma levels of IL-6, a well-established biomarker of systemic inflammation and sepsis morbidity (Remick et al., 2002; Shapiro et al., 2010). IL-6 plasma levels sharply increased after LPS injections and returned to baseline levels by 1 week (Fig. 2*C*). Finally, LPS injection also caused a marked reduction in body weight following the initial dose and steadily decreased with subsequent administrations (Fig. 2*D*). At 24 h post-LPS injection, mice showed an 18% reduction in body weight compared with the control group. The animals’ weight consistently increased beyond 24 h postadministration and reached pretreatment weight 1 week after LPS dosing. Conversely, saline-treated animals showed no differences in mMSS, body temperature, plasma IL-6 levels, or weight throughout the different time points. Mortality rates were <10% in the first 3 d during LPS injection (acute phase), <5% in the subacute (Days 4–7) and chronic (Day 7–1 month) phases, and 0% in saline-injected mice.

10.1523/ENEURO.0426-23.2024.f2-1 Figure 2-1 **Extended data supporting Figure 2. Alterations in each parameter of Murine Sepsis Score (MSS) following LPS injection.** This figure illustrates the changes observed in each individual parameter that contributes to the overall MSS score, subsequent to LPS administration. Parameters such as Appearance, Level of Consciousness, Activity, Response to Stimulus, Respiration, and Nesting behavior are plotted over time to detail the acute (Inj 1 to 72h) and sub-acute (96h to 1 week, wk) effects of LPS-induced systemic inflammation. The mean of individual value±SEM are shown. N=7 Saline, N=13 LPS. Download Figure 2-1, TIF file.

Altogether, the mMSS, body temperature, and IL-6 data indicate that the described model of endotoxemia induced an acute systemic hyperinflammatory response immediately after the first LPS injection, reaching a peak after the third dose and resolving at nearly 72 h after the last injection.

### Acute and chronic functional and molecular alterations in the cerebral microvasculature and neuroinflammation in the endotoxemia-induced systemic inflammation model

**Figure 3.**
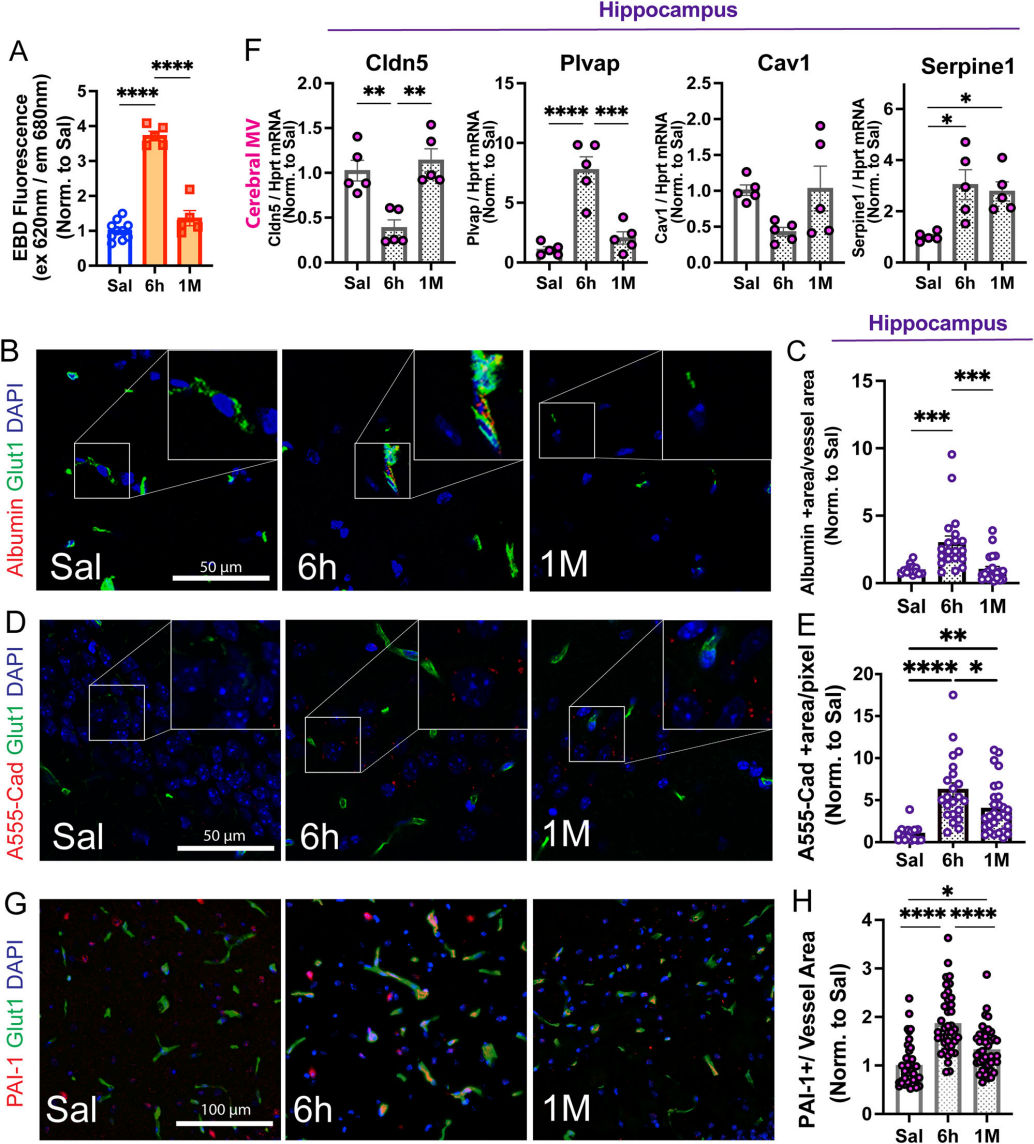
LPS induces acute and chronic alterations in microvascular inflammation and integrity. ***A***, Blood–brain barrier (BBB) permeability, indicated by Evans blue dye (EBD) leakage (albumin), was evident at 6 h post-LPS administration and returned to baseline levels by 1 month after the final LPS injection. Evans blue dye (EBD) fluorescence in brain homogenates normalized to saline is shown. *N* = 5–10 mice/group. ***B–E***, Histopathological validation of BBB leakage in the hippocampus. *N* = 5–8/group. ***B***, ***C***, Albumin leakage in the hippocampus using Alexa Fluor 594 albumin as a tracer shows an increase in albumin-positive area at 6 h following LPS administration that returns to baseline levels at 1 month. ***D***, ***E***, Alexa Fluor 555 cadaverine leakage showed an increase in BBB permeability in the hippocampus 6 h after LPS treatment that remained elevated at 1 month. Panels ***B*** and ***D*** show representative pictures (40× magnification) of the hippocampus with a 2× digital zoom inset. Scale bar, 50 μm. ***F***, LPS-induced systemic inflammation caused a marked reduction in Cldn5 together with an increase in Plvap and the antifibrinolytic Serpine-1 mRNA (which encodes PAI-1), assessed by RT-qPCR in isolated cerebral microvessels (MV). Notice that Serpine-1 mRNA levels remained elevated in the chronic phase (1 month). Fold change LPS compared with saline is plotted. Data points represent the average of duplicates or triplicates of each mouse. Individual values and average ± SEM are shown. *N* = 4–5 mice/group. ***G***, ***H***, Histopathological validation and quantification of PAI-1 alterations (red channel) in the cerebral microvasculature (Glut1-positive areas, green channel) in the hippocampus. *N* = 3 mice/group. Panel ***G*** shows representative pictures of the hippocampus (20× magnification). Scale bar, 100 μm. Nuclear staining (DAPI) is shown in the blue channel in all images. Each data point represents one mouse brain in panels ***A*** and ***F*** and one region of interest (ROI) from the entire scanned image in panels ***C***, ***E***, and ***H***. The individual values and the mean ± SEM are shown. All data were normalized by the mean of saline. *p < 0.05, **p < 0.005, ***p < 0.0005, ****p < 0.0001, one-way ANOVA followed by Tukey's test. Please refer to Extended Data Figure 3-1 for extended data supporting this figure (alterations in microvascular inflammation in cortex and hippocampus) and Extended Data Figure 6-2 for ROI used for quantification of immunofluorescence data.

In order to study the impact of systemic inflammation on BBB function, we used the EBD assay to determine albumin leakage into the brain parenchyma. We observed a robust increase in EBD extravasation in whole-brain homogenates, in the acute phase of systemic inflammation (3.7 ± 0.129-fold, 6 h following the last LPS injection) which returned to baseline levels at 1 month (Fig. 3*A*). To confirm these data histologically and to determine the permeability of macromolecules and small molecules through the BBB in our model, we used two intravascular tracers, Alexa Fluor 594 albumin (∼66 kDa) and Alexa Fluor 555 cadaverine (∼1 kDa), as previously described (Armulik et al., 2010; Knowland et al., 2014; Yanagida et al., 2017). Consistent with the EBD extravasation assay, we observed a robust BBB leakage to Alexa Fluor 594 albumin in the acute phase both in the hippocampus (Fig. 3*B,C*, 3.01 ± 0.48-fold) and cortex (Extended Data Fig. 3-1*A,B*, 2.7 ± 0.23-fold), but not at 1 month. Remarkably, small-molecule (Alexa Fluor 555 cadaverine) leakage was robust in the acute phase and persistent in the chronic phase both in the hippocampus (Fig. 3*D,E*, 6.3 ± 0.8 and 3.9 ± 0.56-fold, respectively) and cortex (Extended Data Fig. 3-1*C,D*). We also analyzed the mRNA levels of molecules governing microvascular function in cerebral microvessels isolated from both control and LPS-treated mice, such as tight junction proteins (Cldn5) and molecules governing vesicular trafficking (Plvap, Cav1) and coagulation (plasminogen activator inhibitor-1, PAI-1, encoded by the transcript Serpine-1). LPS-induced inflammation caused a marked reduction in Cldn5 (∼62% decrease) mRNA levels at 6 h, along with a significant increase in the expression of Plvap (7.8 ± 1.1-fold) and the antifibrinolytic PAI-1 (Serpine-1 transcript, 3 ± 0.58-fold). Interestingly, while the alterations in transcripts governing barrier function reverted to near baseline levels in the chronic phase (1 month after systemic inflammation), the increase in procoagulant Serpine-1 transcript levels was still present at 1 month (Fig. 3*F*, 2.8 ± 0.37-fold). Moreover, the histopathological immunofluorescence (IF) analysis of hippocampal (Fig. 3*G,H*) and cortical (Extended Data Fig. 3-1*E,F*) microvessels using Glut1 antibody as an endothelial-specific marker confirmed the robust increase in the number of PAI-1+ vessels both at 6 h and 1 month (86 and 32% increase, respectively) following LPS administration compared with saline. Since increased levels of PAI-1 contribute to impaired fibrin degradation, fibrin deposition, and microvascular dysfunction in sepsis (Shapiro et al., 2010), we also determined the functional consequences of this sustained PAI-1 upregulation in the cerebral microvasculature by quantifying the levels of fibrin in the cerebral microvasculature in our model. IF analyses with anti-fibrin/fibrinogen and Glut1 antibodies revealed increased intra-/perivascular fibrin/fibrinogen deposition in the chronic phase both in the hippocampus and cortex (1.7 ± 0.1 and 1.8 ± 0.3-fold, respectively, Extended Data Fig. 3-1*G–J*). Altogether, these data indicate that the established endotoxemia model induces cerebral microvascular inflammation (assessed by BBB permeability), expression of procoagulant molecules, and intra-/perivascular fibrin/fibrinogen deposition, both in the acute phase and 1 month after recovery from systemic inflammation.

10.1523/ENEURO.0426-23.2024.f3-1 Figure 3-1 **Extended data supporting Figure 3. Alterations in microvascular inflammation and integrity post systemic inflammation in cortex and hippocampus.** (A-D) Histopathological validation and quantification of BBB leakage in cortex. N=5-8/group. (A, B) Albumin leakage in cortex using Alexa-594 albumin as a tracer shows an increase in albumin+ area at 6h following LPS administration that returns to baseline levels at 1 month. (C, D) Alexa-555 Cadaverine leakage showed an increase in BBB permeability in cortex 6h after LPS treatment that persists at 1 month. Panels A and C show representative pictures (40x magnification) of the cortex with a 2x digital zoom inset. Scale bar=50μm**.** (E, F) Histopathological validation and quantification of PAI-1 alterations (red channel) in the cortical microvessels (Glut1 positive areas, green channel). N=3 mice/group. (G-J) Fibrin/fibrinogen (FBG) immunofluorescence analyses and quantification in intra/perivascular areas (Glut1 positive areas, green channel) in the cortex (G, H) and hippocampus (I, J). N=3 mice/group. Panels E, G and I show representative pictures (20x magnification), along with 2x-zoom insets for panels G and I. Scale bar=100μm. Nuclear staining (DAPI) is shown in the blue channel. (B, D, F, H, J) Each data point represents one region of interest (ROI) from the scanned image. Data are fold induction normalized to the mean of Saline. The individual values and the mean±SEM are shown. *p<0.05, **p<0.005, ***p<0.0005, ****p<0.0001, one-way ANOVA followed by Tukey’s test. Download Figure 3-1, TIF file.

**Figure 4.**
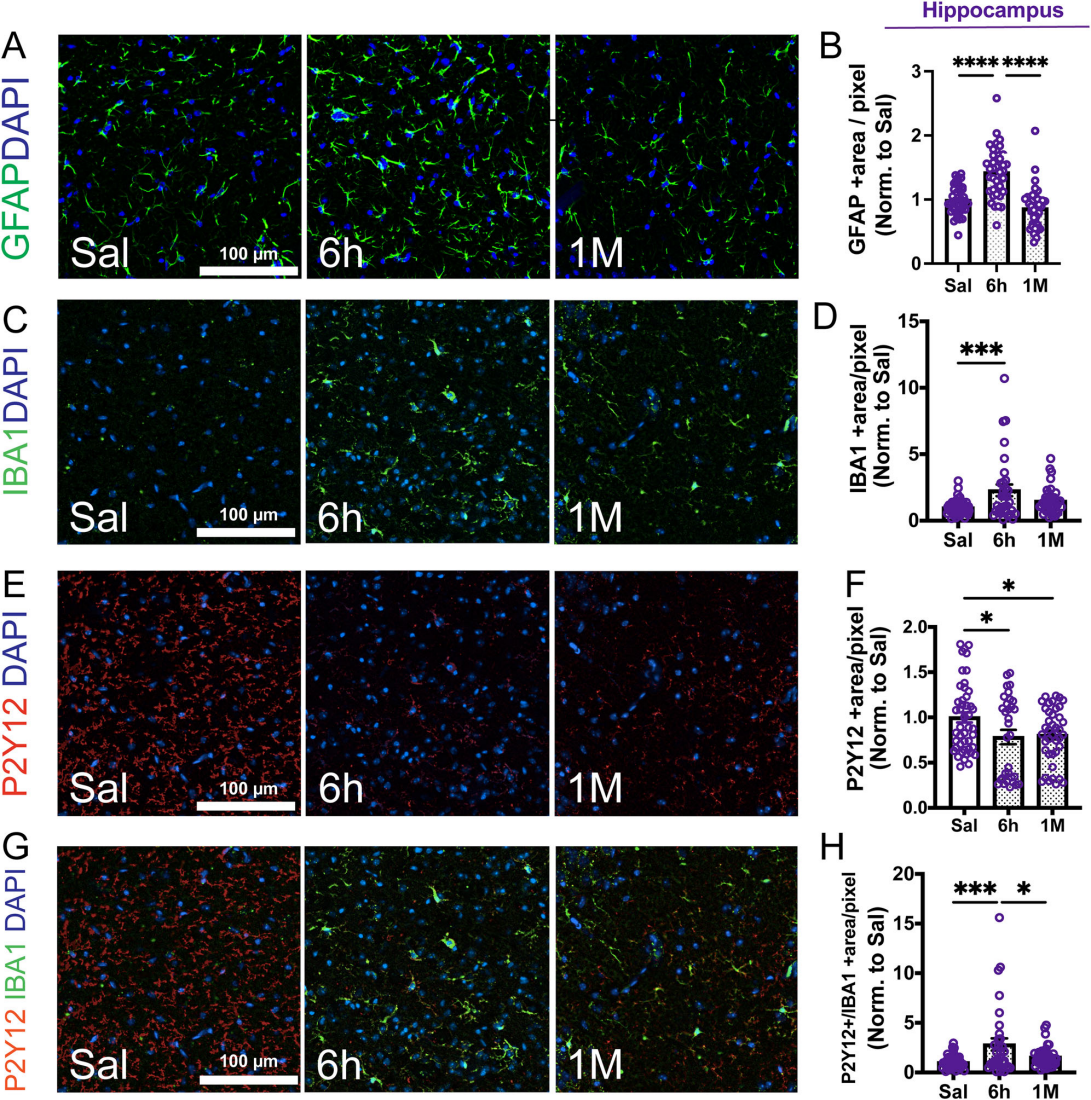
Endotoxin-induced systemic inflammation leads to increased expression of neuroinflammatory markers. Immunofluorescence analyses and quantification for ***A*** and ***B***. Glial fibrillary acidic protein (GFAP, green channel). ***C***, ***D***, Ionized calcium-binding adaptor molecule 1 (IBA1, activated microglia/macrophage marker, green channel). ***E***, ***F***, ADP receptor P2Y12 (P2Y12, homeostatic microglia marker, red channel). ***G***, ***H***, P2Y12/IBA1 (merged) staining in the hippocampus from saline-treated controls and 6 h and 1 month post-LPS administration. ***B***, ***D***, ***F***, ***H***, Quantitative analysis representing the total area of GFAP+, IBA1+, P2Y12+, and IBA1 + P2Y12+ staining in the hippocampus. Notice the transient increase in GFAP and IBA1 immunopositivity at 6 h and the decrease in P2Y12. All nuclei were labeled with DAPI (blue channel). Panels ***A***, ***C***, ***E***, and ***G*** show representative pictures (20× magnification). Scale bar, 100 μm. Each data point symbolizes one region of interest (ROI) from the entire scanned image normalized by the mean of saline. *N* = 3–5 mice per group. The individual values and the mean ± SEM are shown. ****p < 0.0001, one-way ANOVA followed by Tukey's test. Please refer to Extended Data Figure 4-1 for extended data supporting this figure (alterations in neuroinflammatory markers in the cortex) and Extended Data Figure 6-2 for ROI used for quantification of immunofluorescence data.

Next, we quantitatively assessed the levels of neuroinflammatory markers in the cerebral parenchyma following systemic inflammation through IF analyses. We focused on the expression dynamics of GFAP, a canonical indicator for activated astrocytes; the ADP receptor P2Y12, as a marker for microglia (Krasemann et al., 2017; Mildner et al., 2017); and IBA1, an indicator of activated microglia/macrophages. These markers were evaluated in two cerebral regions: the cortex and the hippocampus. Data demonstrate a pronounced increase in GFAP (Fig. 4*A,B*, 1.4 ± 0.06-fold) and IBA1 immunoreactivity in the hippocampus (2.28 ±0.44-fold, Fig. 4*C,D*), together with a modest decrease (∼22%) in the homoeostatic microglial marker P2Y12 (Fig. 4*E,F*), 6 h postfinal LPS administration, underscoring an immediate neuroinflammatory response during the acute phase. Analysis of P2Y12+ and IBA1+ (double positive) immunoreactivity suggested an increase in activated microglia (2.8 ± 0.6-fold, Fig. 4*G,H*). One month after recovery from systemic inflammation, quantification of GFAP and IBA1 alterations revealed a regression of these heightened levels (Fig. 4*A–D*). Interestingly, P2Y12 immunopositivity at 1 month remained significantly lower (∼20%) compared with saline mice, which together with the trend in increased IBA1+/P2Y12+ immunoreactivity (58% increase, *p* = 0.002 by *t* test, 1 month vs saline) suggests continued microglial activation in the chronic phase. Analyses of these alterations in the cortex also followed similar trends (Extended Data Fig. 4-1), suggesting that astrocyte activation surged during the acute phase and then returned to normal levels in the chronic phase, while microglial activation remained elevated, reflected by a 74% increase in IBA1+/P2Y12+ compared with saline. These results suggest a temporal pattern of neuroinflammatory marker expression postsystemic inflammation, with an initial spike in the acute phase and a significant decrease in the chronic phase with lingering, low-grade microglial activation.

10.1523/ENEURO.0426-23.2024.f4-1 Figure 4-1 **Extended data supporting Figure 4. Systemic inflammation induces the expression of neuroinflammatory markers in cortex.** Immunofluorescence images and quantification for (A, B) GFAP, (C, D) IBA1, (E, F) P2Y12 and (G, H) P2Y12/IBA1 staining in the parenchyma of cortex from Saline-treated controls, and 6 hours and 1 month post Saline/LPS administration (all nuclei labeled with DAPI, blue channel). Panels A, C, E and G show 20x representative pictures. Scale bar=100μm. (B, D, F, H) Quantitative analysis representing the total area of GFAP+, IBA1+, P2Y12+ and IBA1+P2Y12+ staining in cortex. Notice the increase in GFAP, IBA-1 and IBA1/P2Y12 immunopositivity and the decrease in P2Y12 in the acute phase, suggesting astrocyte and microglial activation. Each data point symbolizes one region of interest (ROI) from the scanned image and was normalized by the mean of Saline. N=3-5 mice/group. The individual values and the mean±SEM are shown. *p<0.05, **p<0.005, ***p<0.0005, ****p<0.0001, one-way ANOVA followed by Tukey’s test. Download Figure 4-1, TIF file.

In summary, our data indicate that the described LPS model of severe systemic inflammation causes both immediate and lasting molecular and functional alterations in the cerebral microvasculature, as well as neuroinflammation. Key changes in the acute phase include a significant increase in BBB permeability, the acquisition of a procoagulant phenotype in the cerebral microvasculature, and elevated expression of neuroinflammatory markers. This acute neurovascular inflammatory response tends to decrease 1 month after recovery from systemic inflammation. However, BBB permeability to small molecules heightened PAI-1 expression in the cerebral microvasculature, and intra-/perivascular fibrin/fibrinogen deposition was sustained in the chronic phase, alongside persistent microglial activation.

### Impairment in spatial, recognition, and contextual fear memory in the endotoxemia-induced systemic inflammation mouse model

**Figure 5.**
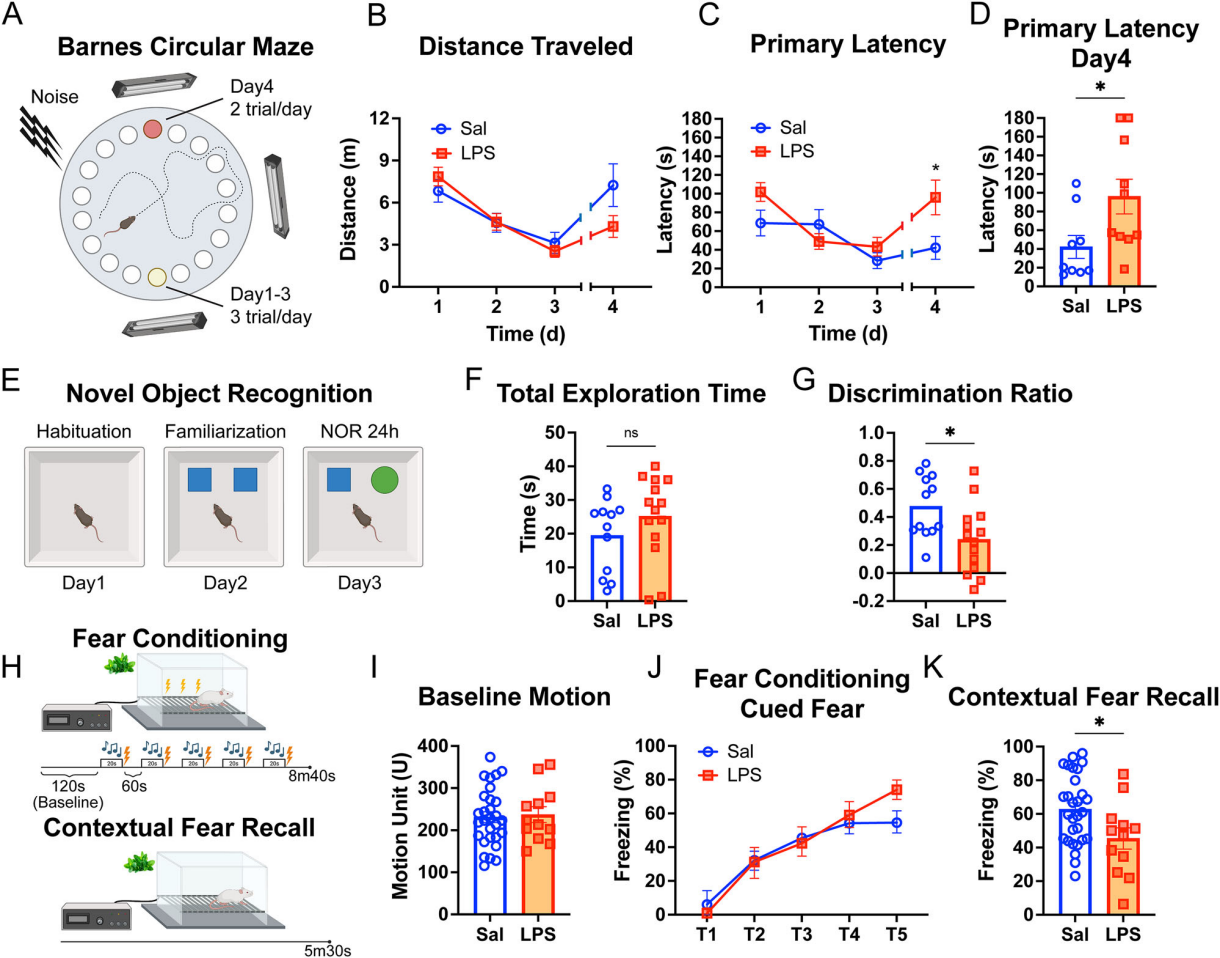
Deficits in spatial, recognition, and contextual fear memory and learning after recovery from systemic inflammation. ***A***, Schematic representation of the Barnes circular maze (BCM) procedures conducted 1 week postrecovery from systemic inflammation. ***B***, Saline- and LPS-injected mice showed similar exploratory behavior assessed by the distance traveled. ***C***, ***D***, LPS-treated mice exhibited spatial memory deficits compared with saline-injected mice. They required more time to locate the escape hole on Day 4 following its relocation (primary latency). ***D***, Individual primary latency values at Day 4 are shown. *p < 0.05, two-way ANOVA followed by Sidak's test or unpaired *t* test. ***E***, Schematic representation of the novel object recognition (NOR) procedures. One month postsystemic inflammation. ***F***, Saline- and LPS-injected mice showed equivalent exploratory movement, assessed by the total exploration time. ***G***, LPS-treated mice show recognition memory deficits compared with saline, failing to recognize the novel object, determined by the lower discrimination ratio. *p < 0.05, unpaired *t* test. ***H***, Schematic representation of the fear conditioning and contextual fear test procedures, conducted 1 month after recovery from systemic inflammation. ***I***, Baseline activity, determined by motion unit automatically calculated by the software, prior to the application of electric shock was comparable between both groups. ***J***, LPS-treated mice did not exhibit deficits in fear learning, assessed by the percentage of freezing time after the tone. ***K***, LPS-treated mice displayed impairments in contextual fear recall compared with saline-injected mice, assessed by the percentage of freezing time after being in the same context but without the tone or application of the electric shock. *p < 0.05, unpaired *t* test (***I***, ***K***) or two-way ANOVA followed by Sidak's test (***J***).

Given the alterations in neurovascular inflammatory markers observed after endotoxemia, particularly in the hippocampus, we aimed next to determine the impact of systemic inflammation on memory and cognition. We performed a series of behavioral tests largely dependent on hippocampal integrity (Bach et al., 1995; D. Y. Chen et al., 2011; Antunes and Biala, 2012; Cohen and Stackman, 2015) to assess memory after recovery from endotoxemia. We conducted testing at Day 7 and 1 month, when mice had recovered from systemic inflammation, and had no visible signs of locomotive or physiological deficits as assessed by the mMSS and plasma IL-6 levels. We performed spatial learning and memory assessment at 1-week postsystemic inflammation using the well-established Barnes circular maze paradigm (Fig. 5*A–D*), the earliest time point when the mice had no visible sign of sickness or locomotive deficits (Fig. 2). Mice were subjected to 3 consecutive training days with three trials per day, with the escape hole located at the same location across trials and days and to two additional trials on Day 4, in which the escape hole was moved to 180° from its previous location. Both saline- and LPS-injected groups exhibited similar locomotive activity with a decrease in the traveled distance from Day 1 to a minimum on Day 3, showing no significant differences between them (Fig. 5*B*). From Day 1 to Day 3 of the test, both groups showed decreasing time of primary latency, which means both groups learned the location of escape hole to the same degree (Fig. 5*C*). Interestingly, when the escape hole was repositioned by 180° on Day 4, as shown in Figure 5*A*, LPS-treated mice required more than twice the time (∼2.3-fold more, *p* < 0.05) to locate the escape hole when compared with saline-treated mice (Fig. 5*C,D*), indicating that they had impaired spatial memory.

Next, we conducted the novel objection recognition (NOR) test to investigate whether our model of sepsis impacts recognition memory in mice 1 month after recovery (Fig. 5*E*, chronic phase). We followed a standard NOR protocol to assess nonspatial recognition memory and followed a 24 h delay between the sample and test sessions. Recognition memory using this paradigm depends on the integrity of both the hippocampus and cortex (Antunes and Biala, 2012; Cohen and Stackman, 2015). On average, there were no significant differences between groups in the total amount of exploration time (Fig. 5*F*). However, LPS-treated mice showed a significantly lower discrimination ratio between novel object and familiar object (∼25%, *p* < 0.05) compared with the saline control group at 24 h postfamiliarization (Fig. 5*G*), indicating that systemic inflammation negatively impacted the ability to remember and recognize the novel object.

To understand the lasting effects of systemic inflammation on emotional memory, we studied mouse performance in fear conditioning and contextual fear recall at 1-month post-LPS administration (Fig. 5*H*). On Day 1, both saline and LPS groups were subjected to fear conditioning by tone test. No differences in baseline motion or percentage of freezing time after each tone were found between groups (Fig. 5*I,J*), indicating that fear learning was not impacted by systemic inflammation. However, when on Day 2, mice from both saline and LPS groups were exposed to the context in which they received the adverse stimulus the day before (LED light and smell of peppermint in the paradigm), LPS-treated mice froze significantly less (∼25%, *p* < 0.05) than the saline group during the first 2 min of the contextual test (Fig. 5*K*). These data indicate that contextual fear memory, which is primarily dependent on hippocampal function (D. Y. Chen et al., 2011), was impaired in mice 1 month after the recovery from systemic inflammation compared with the saline-injected group.

Altogether, these data indicate that the established bacterial endotoxemia model triggers a transient state of systemic inflammation and persistent learning and memory impairment after recovery.

### Alterations in neurofilament, postsynaptic density protein 95, and brain-derived neurotrophic factor expression in the chronic phase after recovery from systemic inflammation

**Figure 6.**
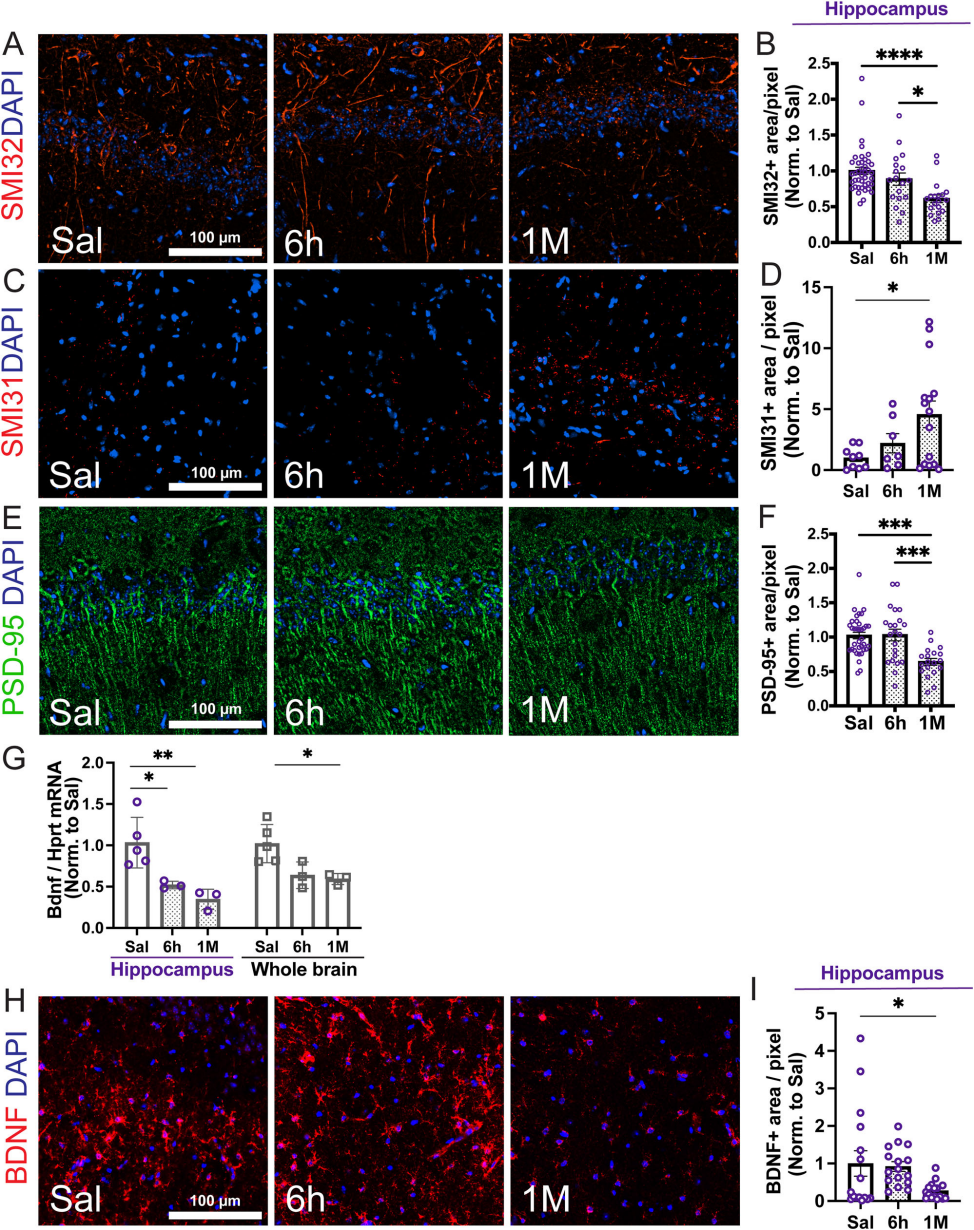
Sepsis induces alterations in neurofilament (NF) phosphorylation, postsynaptic density protein 95 (PSD-95), and brain-derived neurotrophic factor (BDNF) in the hippocampus. Representative immunofluorescence images and quantification for (***A***, ***B***) SMI32 (unphosphorylated NF, red channel), (***C***, ***D***) SMI31 (hyperphosphorylated NF, pNF, red channel), and (***E***, ***F***) PSD-95 (green channel) staining in the hippocampus from saline-treated controls, 6 h (acute phase) and 1 month (chronic phase) post-LPS administrations. Systemic inflammation caused a decrease in SMI32 and PSD-95, together with an increase in SMI31 immunopositivity in the hippocampus. Representative images from the CA1 region are shown. ***G***, Decrease in brain-derived neurotrophic factor (Bdnf) mRNA levels in both the hippocampus and whole brain following systemic inflammation [acute, 6 h, and chronic, 1 month (M) phases]. qRT-PCR analyses of Bdnf mRNA normalized by Hprt are shown. Fold change LPS compared with saline is plotted. Data points represent the average of duplicates or triplicates of each mouse. Individual values and average ± SEM are shown. *N* = 3–5 mice/group. ***H***, Representative immunofluorescence images for BDNF staining (red channel) in the hippocampus from saline-treated controls, 6 h and 1 month after saline/LPS administration. ***I***, Quantitative analysis representing the total area of BDNF+ staining in the hippocampus. ***A***, ***C***, ***E***, ***H***, Representative pictures (20× magnification). Scale bar, 100 μm. Nuclear staining (DAPI) is shown in the blue channel. ***B***, ***D***, ***F***, ***I***, Quantification of the positive SMI32, SMI31, PSD-95, and BDNF areas are shown. Each data point represents one region of interest (ROI) normalized by the mean of saline. *N* = 3–5 mice/group. The individual values and the mean ± SEM are shown. ****p < 0.0001, one-way ANOVA followed by Tukey's test. Please refer to Extended Data Figure 6-1 for extended data supporting this figure (alterations in NF, PSD-95, and BDNF in the cortex) and Extended Data Figure 6-2 for ROI used for quantification of immunofluorescence data.

Given the deficits in spatial, recognition, and contextual fear memory observed after recovery from systemic inflammation, we aimed to identify neuronal molecular alterations reflecting these behavioral outcomes. Since alterations in neurofilament in sepsis patients have been associated with cognitive dysfunction (Ehler et al., 2017, 2019), we first determined the levels of nonphosphorylated and hyperphosphorylated neurofilament (NF) in the brain of control and septic mice using IF analysis. We found that postsepsis cognitive dysfunction was associated with a profound decrease (∼40% reduction) in unphosphorylated NF (Fig. 6*A,B*, SMI-32 immunopositivity) and a robust surge in hyperphosphorylated (p)NF (4.6 ± 0.1-fold, Fig. 6*C,D*, SMI-31 immunoreactivity) in both the hippocampus and cortex (Extended Data Fig. 6-1*A–D*), 1 month after recovery. These alterations in NF have been found to precede neurodegeneration (Wilson et al., 2016; Krasemann et al., 2017) and cognitive decline (Vickers et al., 1994; Bussière et al., 2003; Thangavel et al., 2009) both in aging and Alzheimer's disease. These results suggest the presence of axonal injury in our model and identify NF and pNF as promising neuronal molecular markers of the long-term functional repercussions of severe systemic inflammation.

10.1523/ENEURO.0426-23.2024.f6-1 Figure 6-1 **Extended data supporting Figure 6. Systemic inflammation induces alterations in neurofilament phosphorylation, post-synaptic density 95 (PSD-95) and brain derived neurotrophic factor (BDNF) in cortex.** Immunofluorescence images and quantification for (A, B) SMI32 (unphosphorylated NF, red channel), (C, D) SMI31 (hyperphosphorylated NF, pNF, red channel), (E, F) PSD-95 (green channel) and (G, H) BDNF (red channel) in the cortex of Saline-treated controls, and 6 hours and 1 month post Saline/LPS administrations. Nuclear staining (DAPI) is shown in all images (blue channel). Scale bar=100μm. Notice the decrease in SMI32, PSD-95 and BDNF, together with an increase in SMI31 immunopositivity in cortex after LPS injections. Panels A, C, E and G show representative pictures (20x magnification) and B, D, F and H show the quantitative analyses. Each data point symbolizes one region of interest (ROI) from the scanned sections, normalized by the mean of Saline. N=3-5 mice/group. The individual values and the mean±SEM are shown. **p<0.005, ***p<0.0005, ****p<0.0001, one-way ANOVA followed by Tukey’s test. Download Figure 6-1, TIF file.

10.1523/ENEURO.0426-23.2024.f6-2 Figure 6-2 **Extended data figure supporting figures 3, 4 and 6. ROI used for quantification of histological analyses.** Schematic of the ROI used for quantification. Download Figure 6-2, TIF file.

Synapse loss and reduction of synapse markers have been reported to be hallmark features of memory impairment (Shao et al., 2011). Thus, we examined the levels of postsynaptic density protein 95 (PSD-95), a major postsynaptic scaffold protein that plays a key role in the structural and functional integrity of excitatory synapses (El-Husseini Ael et al., 2002; X. Chen et al., 2008). We found that immunoreactivity for PSD-95 was significantly decreased in the hippocampus (∼36% reduction, Fig. 6*E,F*) and cortex (∼60% reduction, Extended Data Fig. 6-1*E,F*), 1 month after recovery from systemic inflammation. These data suggest that the described systemic inflammation-induced cognitive impairment model leads to synapsis damage and loss.

We also aimed to investigate the alterations in the expression of the neurotrophic factor brain-derived neurotrophic factor (BDNF), which plays a key role in hippocampal neuronal function (Hofer et al., 1990; Heldt et al., 2014). mRNA quantification by qRT-PCR analyses showed a ∼40 and 65% reduction in Bdnf mRNA at 1 month following LPS injection (*p* < 0.05) in the hippocampus and whole brain, respectively (Fig. 6*G*). These results were further validated by histopathological analyses, showing a sustained depletion in the BDNF-positive area in both the hippocampus (72% reduction, Fig. 6*H,I*) and cortex at 1 month (80% decrease, Extended Data Fig. 6-1*G,H*).

Altogether, these data indicate that our model of systemic inflammation-induced cognitive impairment leads to alterations in neurofilament, PSD-95, and BDNF in the chronic phase (1 month after recovery), suggesting the induction of diffuse axonal damage, postsynaptic degeneration, and impaired neurotrophism.

## Discussion

In the current study, we have established and standardized a murine model of sepsis-induced cerebral microvascular dysfunction and cognitive impairment. This model consists of three intraperitoneal injections of bacterial endotoxin (LPS), which cause an acute and rapid systemic hyperinflammatory response that resolves approximately 72 h after the last administration. This acute phase is characterized by functional (albumin and small-molecule BBB leakage) and molecular alterations (induction of PLVAP and PAI-1) in the cerebral microvasculature and neuroinflammation. Remarkably, some of these perturbations persisted after recovery from the systemic inflammatory response (chronic phase), including small-molecule BBB leakage, elevated levels of PAI-1 in cerebral microvessels, peri-/intravascular fibrin/fibrinogen deposition, and microglial activation. In addition, the chronic phase was also characterized by changes in neuronal molecular markers indicative of axonal and synaptic damage (alterations in neurofilaments and PSD-95) and impaired neurotrophism (decreased BDNF levels), which were associated with memory impairment. This standardized model and the identified molecular markers of neurovascular dysfunction will permit future investigations of the mechanisms underlying systemic inflammation-induced cognitive decline, in particular, elucidation of the role of the cerebral microvasculature and its therapeutic potential.

In our investigation, we used the endotoxemia model to recapitulate key clinical features of systemic inflammatory response syndrome (SIRS) and sepsis, such as the generation of an acute systemic hyperinflammatory response via the activation of pathogen recognition receptors and the release of proinflammatory cytokines (Banks et al., 2015; Ho et al., 2015; Seemann et al., 2017; Zhao et al., 2019; Pires et al., 2020). We monitored the progression of the inflammatory response by quantifying the clinical signs of systemic inflammation (mMSS), body temperature, and plasma IL-6 levels, a reliable biomarker of systemic inflammation and sepsis-related morbidity. In our model, this systemic inflammatory response progressed quickly after the first LPS injection, which is in line with previous studies (Seemann et al., 2017) and rapidly resolved 3 d after the last administration. Notably, systemic inflammation-induced chronic lasting effects in the brain beyond recovery, which were reflected in the impaired spatial, recognition, and emotional memory in LPS-treated mice. The hippocampus, in coordination with the cortex, plays a critical role in learning and memory and is particularly vulnerable to acute and chronic neuroinflammation. Sepsis survivors suffer from compromised spatial memory, decreased visual attention, impaired executive functions within the first year, and a persistent reduction in hippocampal and cortical volume up to 2 years after hospital discharge (Gunther et al., 2012; Semmler et al., 2013; Andonegui et al., 2018). Similar results have been reported in rodents, showing impaired learning and hippocampal damage after sepsis (J. W. Lee et al., 2008; Semmler et al., 2008; Basak et al., 2021; Ge et al., 2023). Based on existing evidence and the observed deficits in spatial, recognition, and contextual memory, our results suggest that compromised hippocampal and cortical neuronal function is recapitulated in our model. One limitation of our study was the use of bacterial endotoxin instead of an active pathogen. Nevertheless, SIRS can develop in the absence of a pathogen, for instance, in response to oncotherapies or trauma, causing neurocognitive impairment (Gust et al., 2017), as recapitulated in our model. Another limitation was that we did not monitor changes in the peripheral leukocyte populations since our main objective was to investigate the neurovascular alterations and their relationship with the systemic inflammatory response. For this reason, we phenotypically characterized the kinetics of the systemic response based on the clinical signs (MSS system) and the plasma IL-6 levels, which is a reliable well-established biomarker of systemic inflammation and sepsis morbidity (Remick et al., 2002; Shapiro et al., 2010; Zhang et al., 2013). According to our findings, the described scoring system is a noninvasive and effective way of tracking the systemic inflammatory response, showing kinetics similar to that of IL-6. Despite these limitations, our work has evidenced significant molecular alterations in the cerebral microvasculature and brain which are reflected in the compromised BBB and cognitive function of mice after recovery from systemic inflammation.

Our sepsis/SIRS model caused chronic lasting effects on the brain after the resolution of the systemic inflammatory response which were reflected in memory deficits and molecular changes indicative of diffuse axonal injury, synapse damage, impaired neurotrophism, and neuroinflammation. Alterations in neurofilament, PSD-95, and brain-derived neurotrophic factor (BDNF) were key molecular changes identified in our study that were associated with cognitive impairment 1 month after recovery from systemic inflammation. Neurofilaments are major components of the axonal cytoskeleton. We observed a marked decrease in the levels of the nonphosphorylated NF heavy subunit (NF-H) and an increase in the levels of hyperphosphorylated (pNF-H), which is specifically transported and accumulated in disconnected axons following injury (Shaw et al., 2005; Anderson et al., 2008; Kelberman et al., 2022). As these components are released into the cerebrospinal fluid (CSF) and later into the systemic circulation (Yuan et al., 2012), and because of their easy access and feasibility for sampling across patients, most of the research concerning pNF-H has focused on circulating levels in the blood and CSF as a predictive biomarker of neuronal injury in different pathological conditions, including Alzheimer's disease (Hu et al., 2002; Zurek and Fedora, 2012; Boylan et al., 2013; Gatson et al., 2014; Shaw, 2015; Shibahashi et al., 2016; Darras et al., 2019; Y. Lee et al., 2020). Similar alterations in NF have been described in the brain (Vickers et al., 1994; Bussière et al., 2003; Thangavel et al., 2009) and retina (Wilson et al., 2016); preclinical and clinical data indicate that hyperphosphorylation of neurofilaments precedes degeneration (Wilson et al., 2016) and that decreased immunoreactivity for nonphosphorylated NF in hippocampal and cortical neurons is linked to cognitive impairment in aging and dementia in patients (Vickers et al., 1994; Bussière et al., 2003; Thangavel et al., 2009). In addition to axonal injury, synapse loss and reduction in synapse markers are also hallmark features of postsepsis memory impairment (Huerta et al., 2016) and early dementia (Terry et al., 1991; Shao et al., 2011). Phagocytosis of postsynaptic proteins by activated microglia has been proposed as a cellular mechanism underlying postsynaptic degeneration in dementia (Roy et al., 2020) and neuroinflammatory pathologies (Jafari et al., 2021). In our model, reduction in PSD-95 levels, a major postsynaptic scaffold protein that plays a key role in the structural and functional integrity of excitatory synapses (El-Husseini Ael et al., 2002; X. Chen et al., 2008) along with microglial activation were associated with sepsis-induced cognitive dysfunction. We also found decreased mRNA and protein levels of BDNF in the brain and hippocampus, both in the acute phase and 1 month after recovery from systemic inflammation. Neurotrophic factors play a crucial role in neuronal function and differentiation. BDNF is a major regulator of neuronal growth and synapse plasticity and is closely involved in learning and memory formation (Z-Y. Chen et al., 2006; Soliman et al., 2010; Dincheva et al., 2014; Giza et al., 2018). This neurotrophic factor is expressed both in cortical and subcortical regions, with the highest expression in hippocampal neurons (Hofer et al., 1990; Timmusk et al., 1993; Bathina and Das, 2015). Previous studies have shown that inflammation downregulates BDNF expression, in both rodents and humans (Gibney et al., 2013; Bouvier et al., 2017; Pedard et al., 2021; Yap et al., 2021; Garbossa et al., 2022). At the same time, BDNF limits the neuroinflammatory response in the brain (Wu et al., 2020; Parrott et al., 2021). Although additional research is needed to explore the signaling pathways leading to the changes observed in BDNF, as well as NF and PSD-95, and to clarify the precise functional implications of these changes in our model, our findings have nonetheless highlighted key molecular neuronal markers that are associated with cognitive dysfunction following severe systemic inflammation.

Another key contribution of our study was the identification of functional and molecular alterations in the cerebral microvasculature in our sepsis model during and after recovery from the systemic inflammatory response. We found robust opening of the BBB to plasma proteins (albumin) and small molecules during the acute phase, which coincided with the upregulation of PLVAP, a caveolar protein that plays a key role in nonspecific endothelial transcytosis and vascular leakage (Wisniewska-Kruk et al., 2016; Bosma et al., 2018). In contrast to endothelial cells from peripheral organs, PLVAP expression is actively repressed in the cerebrovascular and retinal endothelium (Daneman et al., 2010). Our data are consistent with recent studies reporting the induction of PLVAP in hypoxic–ischemic and inflammatory pathological conditions and its role in blood–brain and blood–retinal barrier leakage (Shue et al., 2008; Wisniewska-Kruk et al., 2016; Bosma et al., 2018; Munji et al., 2019; Callegari et al., 2023). We postulate that this early opening of the BBB in the acute phase of sepsis allows the leakage of plasma proteins and cytokines (Qin et al., 2007; Beurel and Jope, 2009; Winkler et al., 2017), which initiates microglial activation (Tejera et al., 2019), impairing their homeostatic function (Jafari et al., 2021) and contributing to synapse loss (Huerta et al., 2016), a hallmark of cognitive impairment and early dementia (Terry et al., 1991; Shao et al., 2011). Another important finding was the upregulation of the antifibrinolytic PAI-1 in the cerebral microvasculature in the acute phase. Increased levels of PAI-1 in sepsis impair fibrin degradation and contribute to intravascular fibrin deposition, microthrombus formation, and microvascular dysfunction (Shapiro et al., 2010; Tipoe et al., 2018). In the healthy brain and cerebral microvasculature, PAI-1 is expressed at minimal levels (Sutton et al., 1994; Yamamoto et al., 2005), yet it is induced in stroke (Tjärnlund-Wolf et al., 2012; Callegari et al., 2023) and Alzheimer's disease patients (Melchor and Strickland, 2005; Oh et al., 2014; Angelucci et al., 2023). Remarkably, in addition to these acute changes observed in the cerebral microvasculature, our study revealed key alterations that continued after recovery from the systemic inflammatory response. We found persistent small-molecule (cadaverine) BBB leakage and elevated levels of both PAI-1 mRNA and protein along with increased intra-/perivascular fibrin/fibrinogen deposition in the chronic phase. These novel findings suggest that chronic cerebral microvascular dysfunction after recovery from systemic inflammation, namely, small-molecule leakage and intra-/perivascular coagulation, could further exacerbate neurovascular inflammation, synaptic and axonal degeneration, and the progression of cognitive dysfunction (Zipser et al., 2007; R. M. Liu et al., 2011; Petersen et al., 2018; Griemert et al., 2019; Merlini et al., 2019). In addition, they highlight the therapeutic potential of the endothelium not only in the acute phase but also in the chronic phase after recovery from sepsis. Interestingly, in other pathologies such as Alzheimer's disease (Oh et al., 2014; Angelucci et al., 2023; Eruysal et al., 2023) and neurodegenerative diseases (Zipser et al., 2007; R. M. Liu et al., 2011; Petersen et al., 2018; Griemert et al., 2019; Merlini et al., 2019), BBB leakage (Nation et al., 2019) and increased levels of PAI-1 and fibrinogen have also been found to correlate with cognitive dysfunction and disease severity. Our data indicate that our SIRS/sepsis model faithfully recapitulates key features of cerebral microvascular dysfunction (i.e., BBB leakage and thromboinflammation) in the acute and chronic phases, which are associated with cognitive impairment, providing a validated model to study the contributions of the cerebral microvasculature to sepsis-associated cognitive decline.

In conclusion, the present study provides a standardized and validated animal model for examining the impact of systemic inflammation on cerebral microvascular and cognitive dysfunction. The molecular and functional alterations identified in the cerebral microvasculature suggest the transition to a permeability and procoagulant state, which persisted after recovery from the systemic inflammatory response. Our work has uncovered molecular markers of cerebral microvascular dysfunction in sepsis highlighting promising opportunities for the therapeutic targeting of the endothelium in the acute and chronic phases. This experimental model will permit future investigations of the mechanisms underlying systemic inflammation-induced cognitive dysfunction, such as the role of the cerebral microvasculature, its therapeutic potential, and the identification of novel endothelial therapeutic targets and biomarkers. These future studies will be critical to improve our understanding of the mechanisms by which systemic inflammation affects the development and progression of cognitive impairment and other neurodegenerative conditions, including dementia or Alzheimer's disease, so that novel effective therapies for these conditions can be developed.

## Synthesis

Reviewing Editor: Francisca Bronfman, Universidad Andres Bello Facultad de Medicina Decisions are customarily a result of the Reviewing Editor and the peer reviewers coming together and discussing their recommendations until a consensus is reached. When revisions are invited, a fact-based synthesis statement explaining their decision and outlining what is needed to prepare a revision will be listed below. The following reviewer(s) agreed to reveal their identity: Sunghee Cho.

The study establishes an experimental model that reflects microvascular changes during systemic inflammation, showing molecular and histological changes in BBB in cerebral microvessels and their effect on cognitive dysfunction. Reviewers agreed that the research presented valuable information, but several aspects must be addressed before publication.

General comments,

1- Systemic inflammation can be triggered by stimuli (such as pathogens, LPS, and oxidized/damaged cellular debris). They do not necessarily evoke the same inflammatory responses defined in this study. Thus, the statement of an optimized mouse model of endotoxemia that recapitulates the pathophysiology of systemic inflammation and related cognitive dysfunction is overstretched. The authors argue no inflammation models are available, which is incorrect (10.1089/sur.2016.021, 10.1177/096805199500200206, 10.1186/s40659-023-00469-8, to name a few). Please discuss, first, the limitation of the study and, second, the model present in this study compared to the literature already available.

2-The methods and results section needs polishing; a lot of information is missing from the first, and the latter is confusing when referring to the specific experiments.

Specific comments

1. Describe all the experimental groups in detail in the method section. The groups are not adequately defined. As described in Figure 1, the BBB leakage assay was done in independent groups and evaluated twice.

2. There is no information on the probes used for mRNA detection. They must be added to the methods section.

3. Clarify in the method section which type of tissue was used for each determination. For example, the RNA analysis does not describe what tissue was used. Also, was the PCR performed in duplicate or triplicate? Include which type of sample was analyzed in each determination.

4. The multi-parametric assessment scale is not described in the methods section.

5. Explain how microvessels are being evaluated using immunofluorescence; please indicate the specific markers.

6. Regarding BDNF determination, which brain structure was analyzed is unclear.

7. Indicate or comment on whether the reduction of BDNF mRNA is due to any structural changes. It would be informative to know whether LPS treatment causes CA1 pyramidal neuronal death or reduces dendritic arborization.

8. Concerning BDNF immunohistochemistry, whether the antibody recognizes biologically active mature form, proBDNF, or both is unclear. Please clearly discuss this point. Comment about the validation of this antibody.

9. The second paragraph of the discussion section is very long, making it hard to read; please improve the flow for the reader.

10. Systemic inflammation is not analyzed in the study; only neuroinflammation is. Therefore, the authors should not assume there is systemic inflammation. The severity and duration of systemic inflammation depend on the type of triggers. To be qualified as an optimized systemic inflammation model, the study needs to show how described markers (e.g., PLVAP, BBB leakage, and pNF and BDNF) are related to other systemic inflammatory conditions. Otherwise, the study is limited to describing LPS-induced inflammatory response and functional outcomes. In Figure 4, are Iba1+ cells microglia or monocyte-derived macrophages? Since BBB leakage is evident at 6h but not in 1M, defining the potential origin of Iba1+ cells at 6H from the periphery would be intriguing.

11. Please note that the novel object recognition task is independent of the hippocampus unless methodological changes, such as changing the arena or the object's localization for the test, are made.
