# Supplementary figures and images for: Molecular and Functional Alterations in the Cerebral Microvasculature in an Optimized Mouse Model of Sepsis-Associated Cognitive Dysfunction
Source: eNeuro. 2024 Sep 26;11(9):ENEURO.0426-23.2024. doi: 10.1523/ENEURO.0426-23.2024 (PMC11439565; doi:10.1523/ENEURO.0426-23.2024)

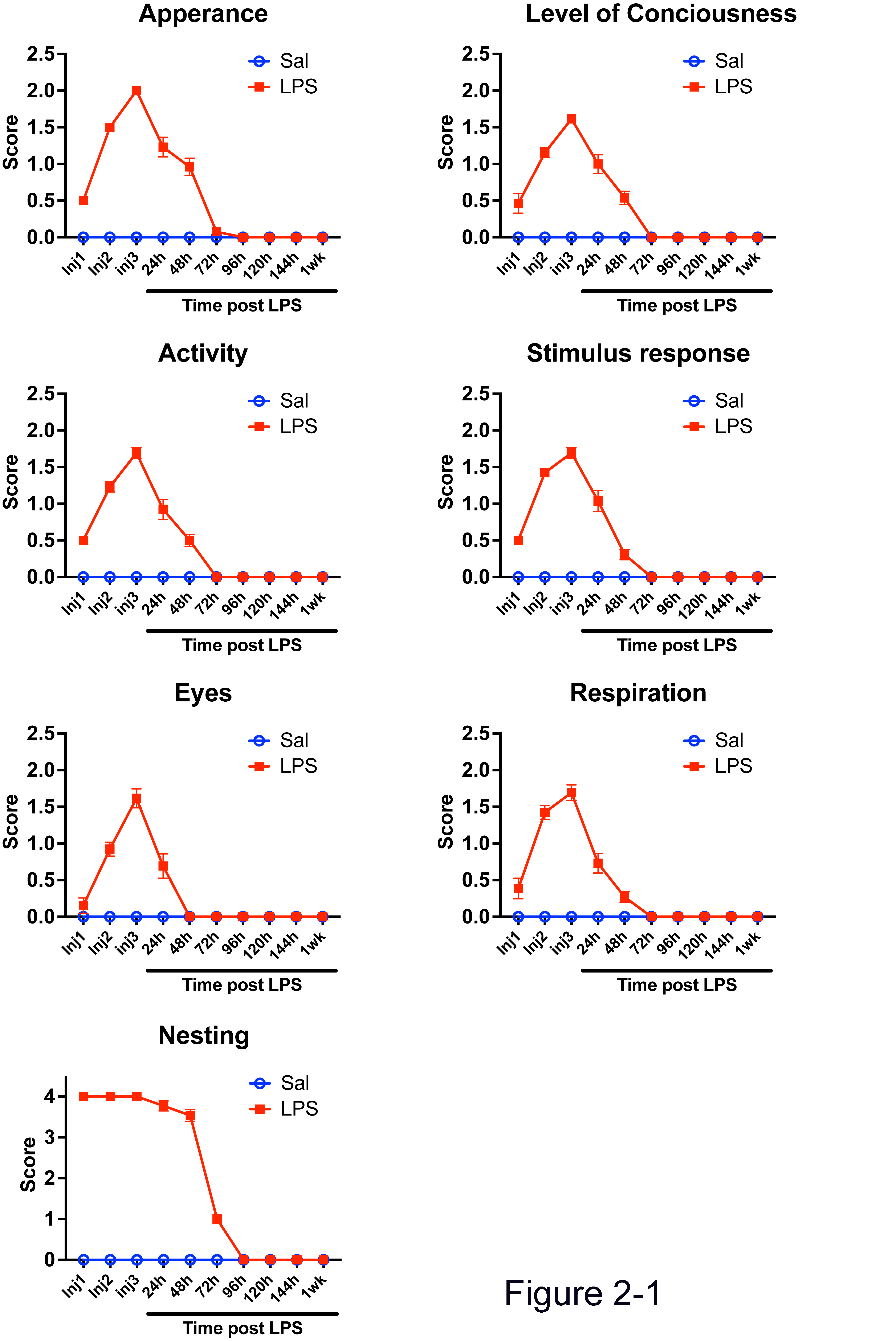

Supplement: Figure 2-1 — Extended data supporting Figure 2. Alterations in each parameter of Murine Sepsis Score (MSS) following LPS injection. This figure illustrates the changes observed in each individual parameter that contributes to the overall MSS score, subsequent to LPS administration. Parameters such as Appearance, Level of Consciousness, Activity, Response to Stimulus, Respiration, and Nesting behavior are plotted over time to detail the acute (Inj 1 to 72h) and sub-acute (96h to 1 week, wk) effects of LPS-induced systemic inflammation. The mean of individual value±SEM are shown. N=7 Saline, N=13 LPS. Download Figure 2-1, TIF file. [file eneuro-11-ENEURO.0426-23.2024-s002.tif]

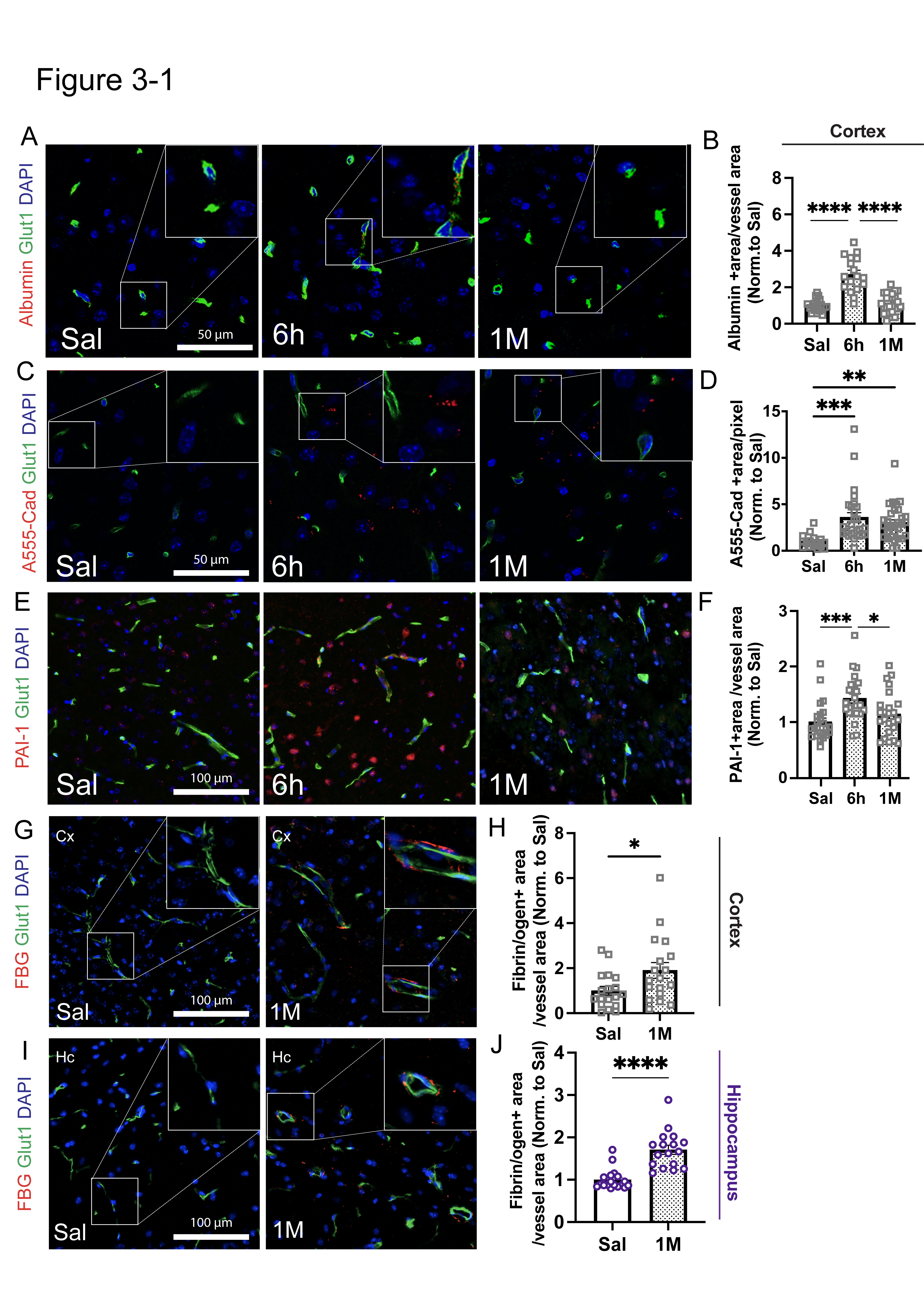

Supplement: Figure 3-1 — Extended data supporting Figure 3. Alterations in microvascular inflammation and integrity post systemic inflammation in cortex and hippocampus. (A-D) Histopathological validation and quantification of BBB leakage in cortex. N=5-8/group. (A, B) Albumin leakage in cortex using Alexa-594 albumin as a tracer shows an increase in albumin+ area at 6h following LPS administration that returns to baseline levels at 1 month. (C, D) Alexa-555 Cadaverine leakage showed an increase in BBB permeability in cortex 6h after LPS treatment that persists at 1 month. Panels A and C show representative pictures (40x magnification) of the cortex with a 2x digital zoom inset. Scale bar=50μm. (E, F) Histopathological validation and quantification of PAI-1 alterations (red channel) in the cortical microvessels (Glut1 positive areas, green channel). N=3 mice/group. (G-J) Fibrin/fibrinogen (FBG) immunofluorescence analyses and quantification in intra/perivascular areas (Glut1 positive areas, green channel) in the cortex (G, H) and hippocampus (I, J). N=3 mice/group. Panels E, G and I show representative pictures (20x magnification), along with 2x-zoom insets for panels G and I. Scale bar=100μm. Nuclear staining (DAPI) is shown in the blue channel. (B, D, F, H, J) Each data point represents one region of interest (ROI) from the scanned image. Data are fold induction normalized to the mean of Saline. The individual values and the mean±SEM are shown. *p<0.05, **p<0.005, ***p<0.0005, ****p<0.0001, one-way ANOVA followed by Tukey’s test. Download Figure 3-1, TIF file. [file eneuro-11-ENEURO.0426-23.2024-s003.tif]

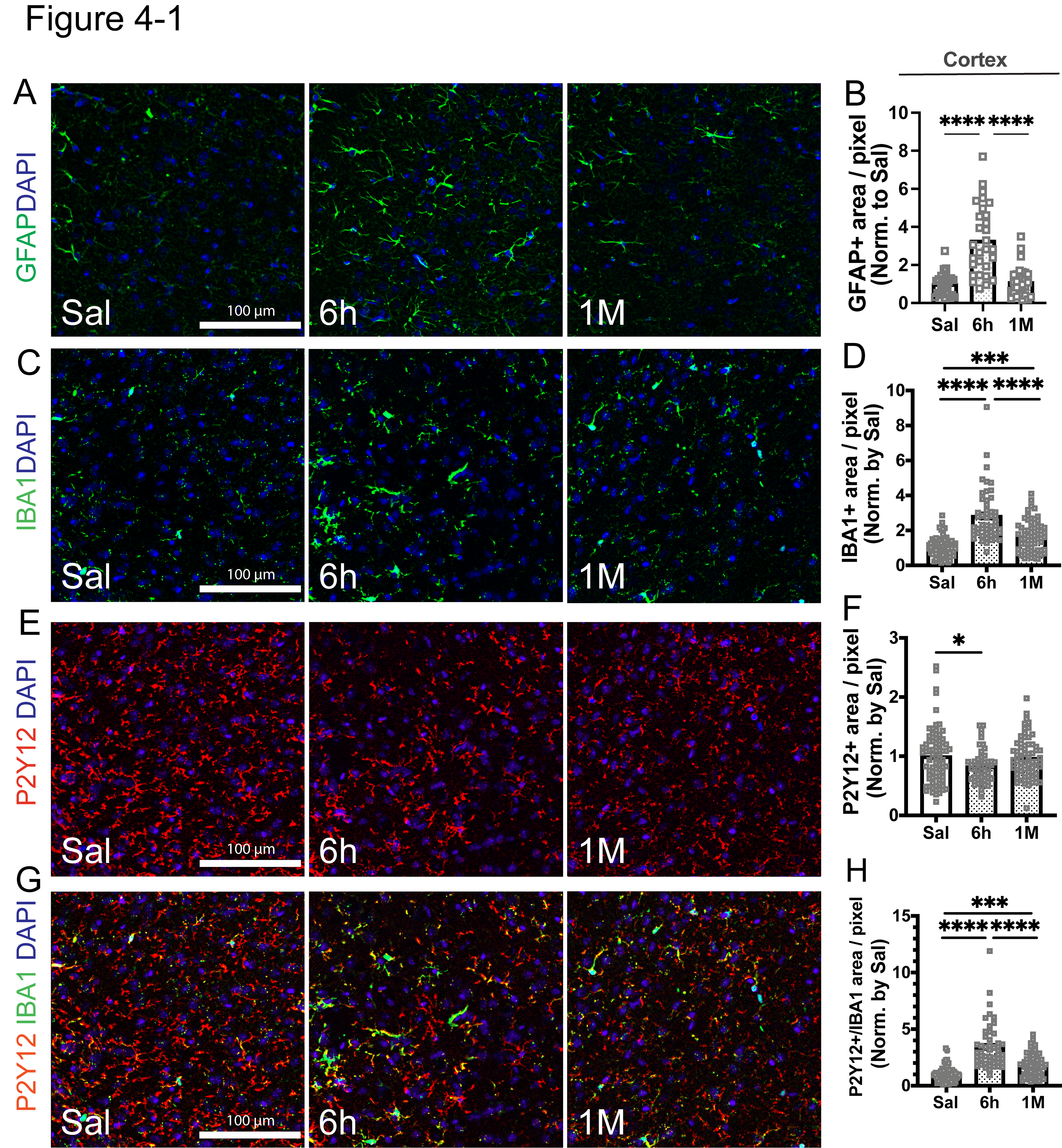

Supplement: Figure 4-1 — Extended data supporting Figure 4. Systemic inflammation induces the expression of neuroinflammatory markers in cortex. Immunofluorescence images and quantification for (A, B) GFAP, (C, D) IBA1, (E, F) P2Y12 and (G, H) P2Y12/IBA1 staining in the parenchyma of cortex from Saline-treated controls, and 6 hours and 1 month post Saline/LPS administration (all nuclei labeled with DAPI, blue channel). Panels A, C, E and G show 20x representative pictures. Scale bar=100μm. (B, D, F, H) Quantitative analysis representing the total area of GFAP+, IBA1+, P2Y12+ and IBA1+P2Y12+ staining in cortex. Notice the increase in GFAP, IBA-1 and IBA1/P2Y12 immunopositivity and the decrease in P2Y12 in the acute phase, suggesting astrocyte and microglial activation. Each data point symbolizes one region of interest (ROI) from the scanned image and was normalized by the mean of Saline. N=3-5 mice/group. The individual values and the mean±SEM are shown. *p<0.05, **p<0.005, ***p<0.0005, ****p<0.0001, one-way ANOVA followed by Tukey’s test. Download Figure 4-1, TIF file. [file eneuro-11-ENEURO.0426-23.2024-s004.tif]

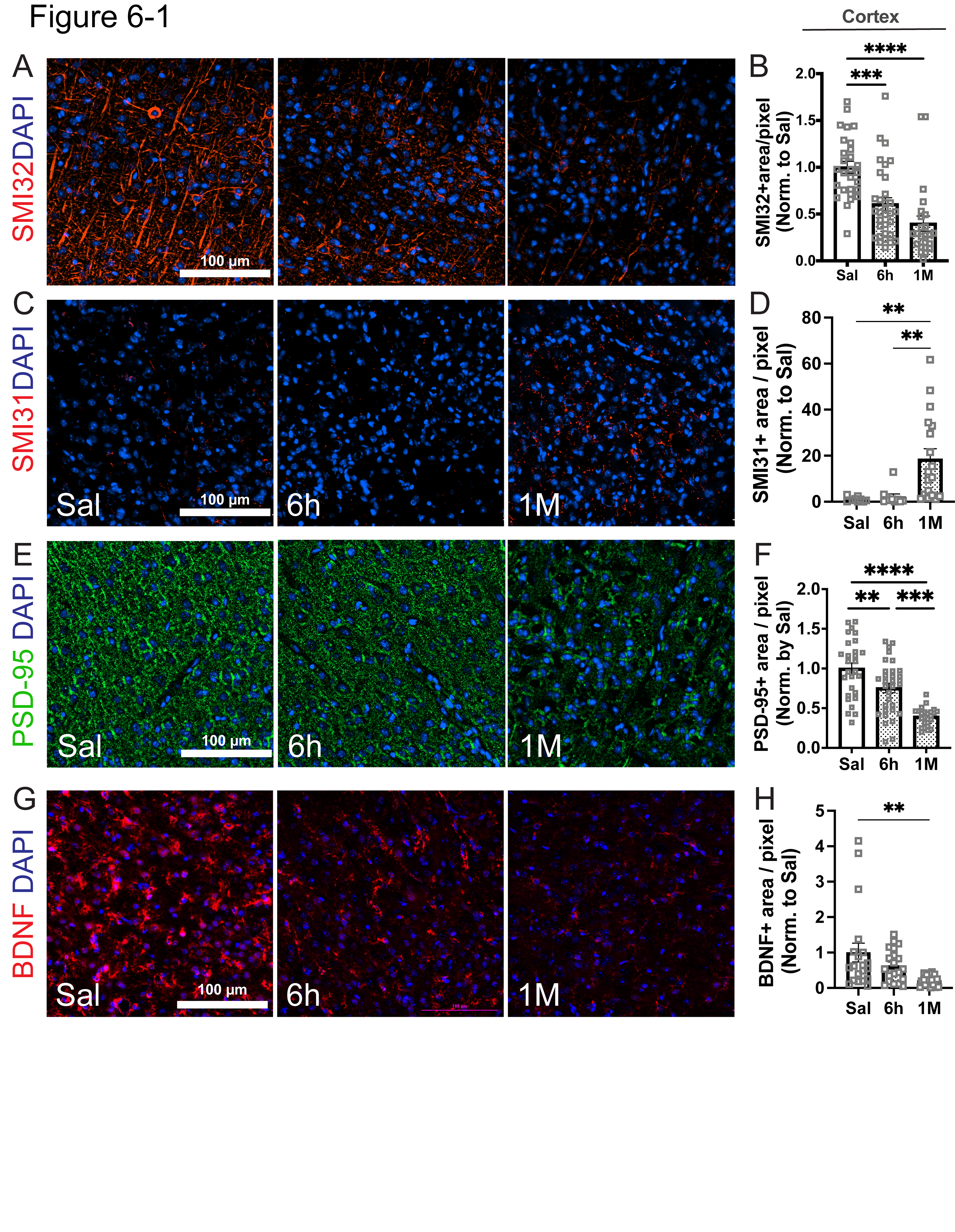

Supplement: Figure 6-1 — Extended data supporting Figure 6. Systemic inflammation induces alterations in neurofilament phosphorylation, post-synaptic density 95 (PSD-95) and brain derived neurotrophic factor (BDNF) in cortex. Immunofluorescence images and quantification for (A, B) SMI32 (unphosphorylated NF, red channel), (C, D) SMI31 (hyperphosphorylated NF, pNF, red channel), (E, F) PSD-95 (green channel) and (G, H) BDNF (red channel) in the cortex of Saline-treated controls, and 6 hours and 1 month post Saline/LPS administrations. Nuclear staining (DAPI) is shown in all images (blue channel). Scale bar=100μm. Notice the decrease in SMI32, PSD-95 and BDNF, together with an increase in SMI31 immunopositivity in cortex after LPS injections. Panels A, C, E and G show representative pictures (20x magnification) and B, D, F and H show the quantitative analyses. Each data point symbolizes one region of interest (ROI) from the scanned sections, normalized by the mean of Saline. N=3-5 mice/group. The individual values and the mean±SEM are shown. **p<0.005, ***p<0.0005, ****p<0.0001, one-way ANOVA followed by Tukey’s test. Download Figure 6-1, TIF file. [file eneuro-11-ENEURO.0426-23.2024-s005.tif]

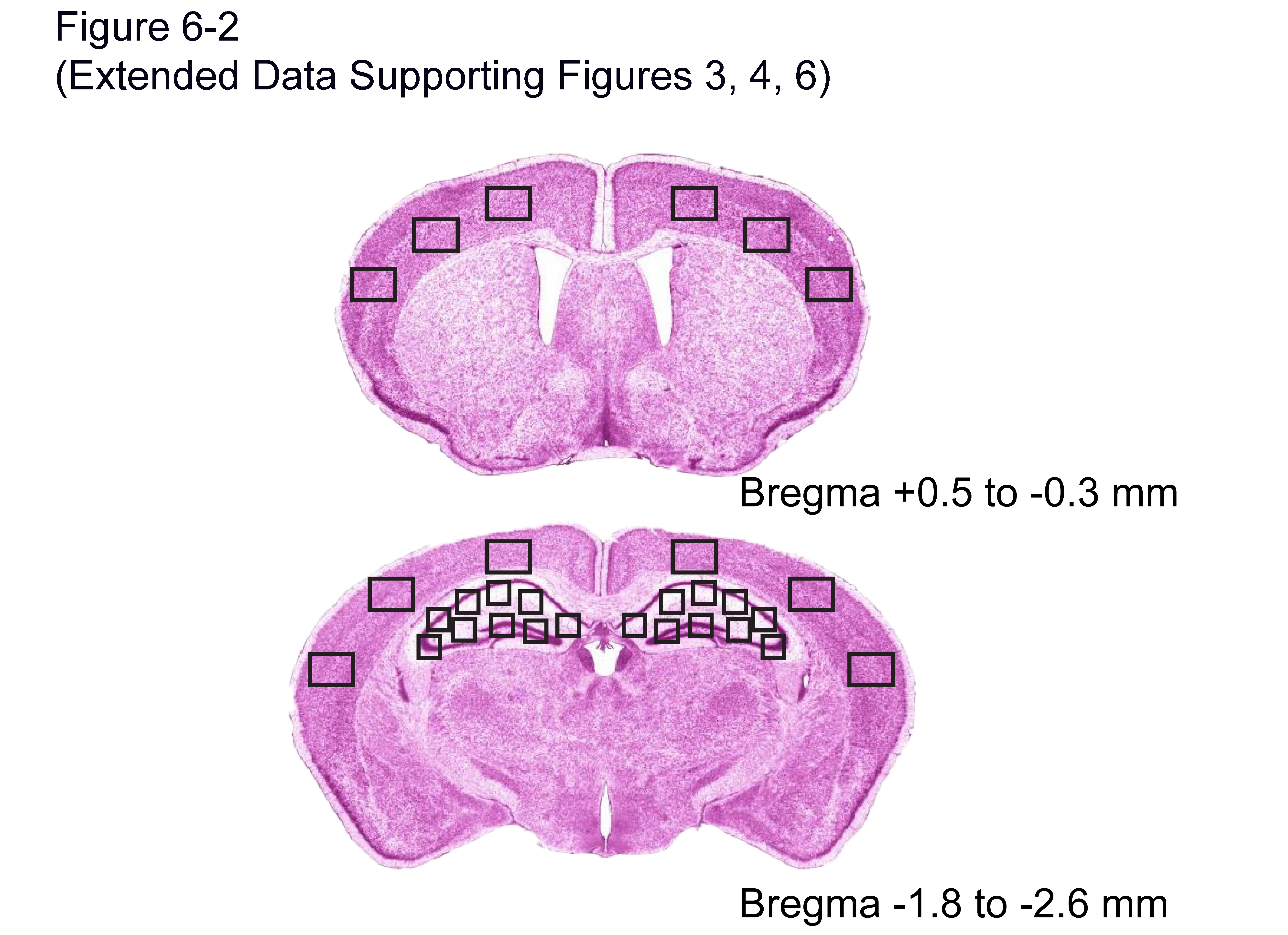

Supplement: Figure 6-2 — Extended data figure supporting figures 3, 4 and 6. ROI used for quantification of histological analyses. Schematic of the ROI used for quantification. Download Figure 6-2, TIF file. [file eneuro-11-ENEURO.0426-23.2024-s006.tif]
